# Natural antidepressants in neuroimmunomodulation: molecular mechanisms, action targets, and therapeutic potential

**DOI:** 10.3389/fimmu.2025.1642001

**Published:** 2025-08-27

**Authors:** Shimeng Lv, Linghui Kong, Xia Zhong, Ruirui Shang, Yitong Lu, Guangheng Zhang, Haonan Gao, Xin Hou, Guoqiang Li, Xiaowen Yu

**Affiliations:** ^1^ First Clinical Medical College, Shandong University of Traditional Chinese Medicine, Jinan, China; ^2^ Institute of Child and Adolescent Health, School of Public Health, Peking University, Beijing, China; ^3^ College of Rehabilitation Medicine, Shandong University of Traditional Chinese Medicine, Jinan, Shandong, China; ^4^ The Second Clinical College of Guangzhou University of Chinese Medicine, Guangdong, China; ^5^ Department of Pain Medicine, The First Affiliated Hospital of Shandong First Medical University & Shandong Provincial Qianfoshan Hospital, Jinan, China; ^6^ Division 4 of Neurology Department, Affiliated Hospital of Shandong University of Traditional Chinese Medicine, Jinan, China

**Keywords:** neuroimmunity, immunoregulation, depression, natural product, molecular biology function

## Abstract

Depression is a major global public health issue, yet key bottlenecks remain in understanding its pathophysiological mechanisms, which significantly hinder breakthroughs in precision treatment strategies. Recent studies have highlighted the neuroimmune system as a primary pathogenic contributor to the onset and progression of depression. Meanwhile, natural products, characterized by multi-component synergy, multi-target activity, and multi-pathway regulation, have shown significant potential in regulating neuroimmunity. However, a systematic review of the role of neuroimmunity in the pathological process of depression and the therapeutic effects of natural products is still lacking. This review aims to comprehensively elucidate the core role of neuroimmunity in the pathological mechanisms of depression through literature analysis, explore in depth the molecular mechanisms and targets involved in natural product interventions, and critically evaluate the limitations and current challenges in clinical translation. Ultimately, this review provides a solid theoretical foundation and guidance for future research and the development of precision antidepressant therapies based on natural products.

## 1 Introduction

Major depressive disorder (MDD), commonly referred to as depression, is one of the leading causes of disability worldwide. Its main features include significant physiological symptoms such as fatigue, weight loss, and decreased appetite. Currently, approximately 300 million people are affected by MDD globally, and its prevalence continues to rise, posing a significant public health burden (1). However, the underlying pathological and physiological mechanisms of MDD remain incompletely understood, which severely limits progress in developing precision treatment strategies. At present, first-line clinical treatment mainly relies on selective serotonin reuptake inhibitors, but these medications are often accompanied by adverse effects such as nausea. In addition, they often have a delayed onset of action, and the non-response rate remains relatively high (2, 3). Therefore, an in-depth analysis of the pathological mechanisms of MDD and the development of new antidepressants with rapid onset, sustained efficacy, and improved safety profiles has become a pressing and central challenge in the field of psychopharmacology.

The immune system plays a central role in the pathophysiological processes of MDD. Immune cells and the cytokines they secrete interact with the brain through the neuro-endocrine-immune network, influencing mood, cognition, and behavioral functions. The neuroimmune system contributes to the progression of depression through multidimensional mechanisms; Therefore, targeting neuroimmune regulation has become one of the key strategic directions in the development of novel antidepressant therapies (4–6). Natural products refer to bioactive substances derived from a wide range of sources, including animals, plants, insects, microorganisms, marine organisms, and endogenous substances in humans and animals. For decades, natural products and their derivatives have been widely studied in the context of health promotion and disease intervention due to their structural diversity, favorable safety profiles, and cost-effectiveness. Among them, numerous natural bioactive molecules have been shown to exert significant immunomodulatory effects (7, 8).

Recent advances in molecular biology have significantly accelerated research into the neuro-immunomodulatory mechanisms of depression, leading to a growing body of compelling evidence. Within this context, natural products have demonstrated unique and promising therapeutic potential in antidepressant strategies due to their characteristic multi-target, synergistic modulation. However, current research still faces critical challenges, including fragmented mechanistic understanding, an unclear network of action targets, and limited clinical translation. These issues severely hinder the development of novel natural antidepressants targeting neuroimmune pathways. This review aims to integrate the existing literature, systematically clarify the central role of the neuroimmune axis in the pathophysiology of depression, and summarize the molecular mechanisms by which natural compounds modulate neuroimmunity. In addition, it critically evaluates the limitations of current research. Ultimately, this review provides a theoretical foundation and offers direction for future research and the clinical translation of neuroimmune-targeted natural antidepressants.

## 2 Review method

To investigate the pathological mechanisms of depression mediated by neuroimmunity and the neuro-immunomodulatory effects of natural products in treating depression, we conducted a comprehensive search of the PubMed, Web of Science, and ScienceDirect databases based on the PRISMA. The keywords used were “natural product,” “depression,” “major depressive disorder,” “neuroimmunity,” “neuroimmune modulation,” “antidepressant,” “neuroinflammation,” “proinflammatory cytokine,” “NLRP3 inflammasome,” “glial cells,” “microglia,” “astrocyte,” “microbial-gut-brain axis,” “programmed cell death,” “pyroptosis,” “autophagy,” “mitochondrion,” “neuroplasticity,” “synaptic plasticity,” “neurogenesis,” “epigenetics,” and “circadian rhythm.” Retrieve articles published from January 2000 to Jun 2025. The retrieved articles were reviewed by two independent reviewers based on their title, abstract, and full text, adhering to inclusion and exclusion criteria. The inclusion criteria included: (1) original articles written in English; (2) the article investigates the mechanisms by which natural products regulate neuroimmune therapy for depression. Exclusion criteria include: (1) articles written in any language other than English; (2) gray literature; (3) editorials; and (4) duplicate publications. After completing the literature search and screening process, a total of 227 articles were included in the final evaluation.

## 3 Neuroimmunity and depression

### 3.1 Immune dysregulation in depression

There is ample evidence suggesting the presence of peripheral and neuroimmune dysregulation in patients with depression. In the brains of suicidal patients with depression, upregulation of NOD-like receptor family pyrin domain containing 3 (NLRP3) inflammasome expression has been observed (9). In patients with MDD, genes related to endoplasmic reticulum stress (ERS) and inflammasomes (such as NLRC4 and NLRP3) are significantly upregulated, suggesting that the enhancement of immune-inflammatory mechanisms in MDD may be closely related to the synergistic effects of organelle dysfunction (involving the endoplasmic reticulum and mitochondria) and inflammasome activation (10). Elevated levels of interleukin-6 (IL-6) and interleukin-8 (IL-8) in MDD patients, as well as abnormal functional connectivity between the prefrontal cortex (PFC), anterior cingulate cortex (ACC), visual cortex, postcentral gyrus, and striatum, suggest that inflammation may contribute to the neuropathological mechanisms of MDD by altering the functional connectivity of key brain regions such as the PFC (11). Moreover, pro-inflammatory cytokines, including tumor necrosis factor-alpha (TNF-α) and IL-1β, serve as important indicators reflecting systemic inflammatory status, offering objective and indispensable measures for evaluating the degree of immune activation in patients with MDD (1). In clinical studies, Li et al. found that the volume of gray matter in the right frontal gyrus is a key mediating factor between IL-1β levels and antidepressant response. IL-1β may indirectly reduce treatment efficacy by decreasing the volume of this brain region (12).

In addition, patients with MDD exhibit increased choroid plexus volume and decreased lymphatic system function, which are closely related to systemic inflammation and oxidative stress, and may contribute to the pathological process of MDD through immune mechanisms (13). In a recent cross-sectional study based on the National Health and Nutrition Examination Survey in the United States, it was found that the aggregate index of systemic inflammation (AISI) level has a U-shaped correlation with depression, and maintaining AISI within a reasonable range may help reduce the incidence of depression (14). A study investigating sex differences in patients with depression revealed that the association between inflammation and depression differs by gender, with female patients more likely to exhibit elevated levels of C-reactive protein and IL-6 (15). Systemic inflammation, as indicated by elevated IL-6 levels, may affect the severity and progression of depressive symptoms, especially in adolescent women (16).

Overall, there is a significant correlation between depression and immune homeostasis imbalance. Patients with depression exhibit immune dysregulation in both peripheral blood and the central nervous system (CNS). Further analysis of the key molecular mechanisms involved in neuroimmune interactions, particularly the elucidation of the biological effects of specific inflammatory mediators within the CNS, will help overcome the limitations of traditional antidepressant treatments that primarily target monoamine neurotransmitters. This will provide an important theoretical foundation for the development of precision treatment strategies based on immune regulation.

### 3.2 Neuroimmune mediated pathological mechanism of depression

#### 3.2.1 Cytokine

In depression, there is a pathological phenomenon characterized by the upregulation of pro-inflammatory cytokines, as evidenced by a meta-analysis showing a significant increase in pro-inflammatory cytokines in patients with MDD (17). However, the potential molecular mechanisms underlying this phenomenon have not yet been fully elucidated.Interleukin-33 (IL-33), a tissue-derived nuclear cytokine from the IL-1 family, has been implicated in this process (18). Studies suggest that IL-33 may disrupt the coordination between hippocampal metabolic rhythms and circadian clock genes by activating inflammatory signaling and interfering with mitochondrial metabolism, ultimately leading to depression-like behavior (19). In addition, interleukin-1β (IL-1β) plays a significant regulatory role in the connection between the mid-frontal gyrus and the middle cingulate cortex/insula junction and depressive symptoms, suggesting that inflammation may serve as a bridge linking brain dysfunction and depressive symptoms (20). An imbalance between IL-1 receptor antagonist (IL-1ra) and IL-1β reduces the expression of the cAMP response element-binding protein (CREB)-brain-derived neurotrophic factor (BDNF) in the hippocampus and disrupts neurotransmission mediated by α-amino-3-hydroxy-5-methyl-4-isoxazolepropionic acid receptors (AMPARs), resulting in depression-like behavior (21). Knockout of IL-1β in the hippocampus significantly reduces lipopolysaccharide (LPS)-induced anxiety and depression-like behavior. IL-1β knockdown also inhibits oxidative and neuroinflammatory responses and upregulates vascular endothelial growth factor and BDNF levels in the hippocampus (22). Another essential pro-inflammatory cytokine involved in immune modulation is interleukin-18 (IL-18) (23). It has been reported that IL-18-deficient mice are more susceptible to stress, exhibiting significantly increased expression of inflammatory factors (TNF-α, IL-1β, and IL-6) in the hippocampus, along with increased numbers of activated microglia and astrocytes (24). During antidepressant treatments, such as Integrative Body-Mind-Sleep and Qigong interventions, significant reductions in depressive symptoms, sleep disturbances, and levels of IL-6 and IL-1β have been observed (25). A comprehensive analysis of existing evidence indicates that pro-inflammatory cytokines play a key regulatory role in the pathological progression of depression. Building upon this foundation, further exploration of the upstream molecular mechanisms regulating their release (such as the activation of the NLRP3 inflammasome pathway) may provide crucial insights into the multifaceted molecular processes underlying neuroimmune dysregulation in depression.

#### 3.2.2 NLRP3 signaling pathway

A cytoplasmic multiprotein complex known as the NLRP3 inflammasome responds to external environmental stress and cellular injury. Upon activation, the assembled NLRP3 inflammasome promotes the maturation and release of inflammatory cytokines, including IL-18 and IL-1β, by activating caspase-1 (26). NLRP3 is closely associated with depression. Chronic mild stress (CMS) activates the NLRP3 inflammasome, enhances the maturation and release of IL-1β, and subsequently triggers neuroinflammation, ultimately leading to depression-like behavior (27). Furthermore, the NLRP3 inflammasome contributes to LPS-induced depression-like behavior through the induction of indoleamine 2,3-dioxygenase (28). It is also involved in chronic unpredictable mild stress (CUMS)-induced Alzheimer’s disease-like pathological changes and related cognitive impairment (29). In addition, NLRP3 mediates the activation of inflammasomes, leading to depression-like behaviors caused by estrogen deficiency and hippocampal inflammation in mice (30). The expression of proteins related to the NLRP3 inflammasome pathway is significantly upregulated in the ischemic hippocampus of post-stroke depression (PSD) mice. However, when the hyperpolarization-activated cyclic nucleotide-gated cation channel 1(HCN1) was knocked out by injecting a virus into the hippocampus, the expression of NLRP3 inflammasome-related proteins in PSD mice decreased, and both anxiety- and depression-like behaviors were alleviated (31).

On the other hand, NLRP3 also mediates associated signaling pathways involved in the pathology of depressive symptoms. One key purinergic receptor that plays a central role in inflammation and immunity is the P2X7 receptor (P2X7R), which is expressed in almost all cells of the innate and adaptive immune systems. P2X7R mediates various key biological processes (32). In depression, rats exposed to CUMS exhibit upregulation of the P2X7R/NLRP3/IL-1β signaling axis and display depression-like behaviors (33). Moreover, CUMS activates P2X7R in microglia, triggering neuroinflammatory responses through the activation of the NLRP3 inflammasome and the release of inflammatory molecules such as IL-1β (34). Activation of P2X7R and the downstream NLRP3 inflammasome in hippocampal microglia also mediates depression-like behavior (35). Although psychological stress is known to contribute to emotional disorders such as depression, the underlying molecular mechanisms require further elucidation. Recent studies by Li et al. demonstrated that psychological stress activates the P2X7R/NLRP3 inflammasome pathway, leading to amygdala demyelination and oligodendrocyte dysfunction, ultimately resulting in emotional disturbances, particularly depression (36). These findings highlight the critical role of the P2X7R/NLRP3 signaling pathway in emotional disorders, especially depression. Therapeutically targeting this pathway, for example, through transcutaneous auricular vagus nerve stimulation, can suppress microglia-mediated neuroinflammation and alleviate diabetes-related depression induced by a high-fat diet via modulation of the P2X7R/NLRP3/IL-1β signaling pathway in the prefrontal cortex (37). In addition, NLRP3 mediates other signaling pathways relevant to depression. For example, in CUMS-induced depression-like behavior in mice, activation of the ERS-NLRP3 signaling pathway contributes to both depressive behavior and cognitive dysfunction (38). Silent Information Regulator 2 Homolog 1 (SIRT1), a member of the nicotinamide adenine dinucleotide (NAD^+^)-dependent deacetylase family, is closely associated with the onset and progression of inflammation-related disorders through the SIRT1/NLRP3 signaling pathway (39). Recent studies indicate that nicotinic acid may exert a protective effect against LPS-induced depression-like behavior in mice by modulating the SIRT1/poly (ADP-ribose) polymerase-1 (PARP-1)/NLRP3 signaling cascade (40). Melatonin alleviates LPS-induced acute depression-like behavior and suppresses NLRP3 inflammasome activation in microglia via the SIRT1/nuclear factor erythroid 2-related factor 2 (Nrf2) pathway (41).

In summary, the NLRP3 inflammasome and its associated inflammatory signaling pathways play significant roles in both the pathogenesis and potential therapeutic intervention of depression. Further elucidation of the specific molecular mechanisms underlying glial cell-mediated neuroinflammation will enhance our understanding of the pathophysiological mechanisms of CNS in depression from a multidimensional perspective.

#### 3.2.3 Microglia

Microglia are resident macrophages of the brain that play a vital role in immune surveillance and the maintenance of CNS homeostasis. They are functionally implicated in various cerebrovascular diseases by regulating neuroinflammatory responses and facilitating tissue repair processes (42). An integrated spatial and mononuclear transcriptomic analysis revealed that gene changes related to depression-like behavior were predominantly localized in microglia. In particular, a pro-inflammatory microglial subpopulation was identified, characterized by the expression of genes involved in neuroinflammation and potentially contributing to the pathology of depression through the activation of inflammatory pathways such as nuclear factor kappa B (NF-κB) (43). In the ACC, the innate immune receptors known as triggering receptors expressed on myeloid cells-1 and -2 (TREM-1/2) in microglia have been shown to mediate visceral hypersensitivity and depression-like behavior after colitis (44). Furthermore, early-life inflammation can impair the phagocytic capacity of microglia, potentially leading to maladaptive responses of ACC glutamatergic neurons to stress, thereby promoting the emergence of depression-like symptoms during adolescence (45). When early-life stress is combined with heightened cortisol reactivity, it may further exacerbate neuroimmune disturbances in adulthood. This is evidenced by increased microglial activation in the hippocampus and elevated expression of TNF-α and microRNA-342 (miR-342) (46).

Exposure to chlorpyrifos induces a primed state in microglia, rendering them more susceptible to subsequent stress, which then promotes their inflammatory activation. This sequence leads to impaired neural plasticity and the development of depression-like behavior in the hippocampus (47). Additionally, there is growing evidence that the NLRP3 inflammasome plays a pathogenic role within microglia. Specifically, activation of the glucocorticoid receptor (GR)-NF-κB-NLRP3 signaling pathway in hippocampal microglia has been shown to mediate neuroinflammation and promote depression-like behaviors under chronic stress conditions (48). Furthermore, Pan et al. demonstrated that IL-1β-related inflammation in the prefrontal cortex of depressed rats is mediated by microglial NLRP3 inflammasome activation in response to chronic stress (49). Similarly, depression-like behavior induced by streptozotocin (STZ) in diabetic mice may be associated with activation of the NLRP3 inflammasome, primarily within hippocampal microglia (50). In addition, retinal injury may initiate hippocampal microglial activation via the NLRP3/IL-1β pathway, leading to neuroinflammation and subsequent depression-like behavior (51).

Targeting microglia regulation offers promising therapeutic strategies for alleviating depressive-like behaviors. For instance, poly (ADP-ribose) polymerase 14 (PARP14) has been shown to inhibit microglial activation by promoting reactive oxygen species (ROS) clearance through nicotinamide nucleoside transhydrogenase (NNT), thereby reducing depression-like behavior in mice (52). Minocycline, a tetracycline antibiotic with broad anti-neuroinflammatory properties, readily crosses the blood–brain barrier (BBB) and inhibits the activation of pro-inflammatory microglia, exerting neuroprotective effects (53). In depression models, minocycline alleviates depression-like symptoms by blocking microglial activation and phagocytosis, which helps restore neurogenesis in the dorsal hippocampus (54). Furthermore, Forkhead box O3a (FOXO3a) regulates microglial phenotype by suppressing peroxisome proliferator-activated receptor gamma (PPAR-γ). Inhibition of FOXO3a can polarize microglia from the pro-inflammatory M1 phenotype to the anti-inflammatory M2 phenotype, thereby reducing hippocampal neuroinflammation and ameliorating depressive-like behaviors in LPS-induced mouse models (55).

#### 3.2.4 Astrocyte

Astrocytes are the most abundant glial cells in the CNS and perform a variety of essential homeostatic functions *in vivo*. These include supporting other CNS-resident cells, such as neurons, by buffering excess neurotransmitters and regulating synaptic activity and BBB integrity (56). In patients with MDD, astrocyte derived extracellular vesicles exhibit significantly elevated levels of pro-inflammatory cytokines such as IL-6, TNF-α, and IL-1β, indirectly reflecting the involvement of astrocytes in depressive neuropathology (57). Moreover, during depression, bidirectional interactions occur between astrocytes and microglia. Chronic social defeat stress (CSDS) induces microglial activation of P2X7Rs and subsequent IL-6 release, which binds to IL-6 receptors on astrocytes, triggering apoptotic pathways, reducing astrocyte numbers, and impairing their function—contributing to anxiety- and depression-like behaviors (58). In astrocytes lacking the sigma-1 receptor, the NF-κB inflammatory pathway is activated. Interactions between reactive astrocytes and activated microglia further amplify neuroinflammation and exacerbate stress-induced neuronal damage (59). In a mouse model of sepsis-associated encephalopathy, astrocytic A1 adenosine receptors mediate the pro-inflammatory effects of adenosine, promoting microglial activation, BBB disruption, peripheral immune cell infiltration, neuronal dysfunction, and depression-like behaviors (60). In astrocyte-specific Orai1 knockout mice, Orai1 deficiency significantly suppresses LPS-induced hippocampal cytokine release and microglial activation, while also preventing calcium signaling enhancement and metabolic disturbances in astrocytes (61). Additionally, in astrocyte-specific NR2C knockout mice, NR2C deficiency preserves the activity of the phosphatidylinositol 3-kinase (PI3K)/protein kinase B (Akt)/mechanistic target of rapamycin (mTOR) pathway, inhibits downstream NF-κB signaling, and maintains synaptic protein expression and dendritic spine integrity, thereby reversing LPS-induced synaptic damage (62). Furthermore, LPS stimulation increases Kir4.1 expression in astrocytes, enhances N-methyl-D-aspartate receptor activity via GluN2B phosphorylation, activates calpain-1 through calcium influx, and triggers the NLRP3 inflammasome, leading to IL-1β release, impaired synaptic plasticity, and depression-like behaviors (63).

In addition, several astrocyte-specific molecular mechanisms have been implicated in the regulation of neuroinflammation and the development of depression. The multiple endocrine neoplasia type 1 (Menin) gene in astrocytes regulates neuroinflammation by inhibiting the NF-κB/IL-1β pathway; functional deficiencies or genetic mutations in Menin may contribute to the onset of depressive disorders (64). The β-arrestin2-biased signaling pathway downstream of dopamine receptor D2 is significantly disrupted during the progression of depression. Notably, genetic deletion of β-arrestin2 exacerbates neuroinflammatory responses and promotes depression-like behaviors (65). Furthermore, neural precursor cell expressed, developmentally downregulated 4 (NEDD4)-like E3 ubiquitin protein ligase (NEDD4L) regulates P2X7R expression by ubiquitinating paired box 6, thereby modulating astrocyte function and neuroinflammatory signaling (66).

In summary, the glial cell system demonstrates a complex molecular network involved in the neuroimmunomodulation of depression. In particular, molecular interactions between microglia and astrocytes play a pivotal role in the neuroimmune pathogenesis and therapeutic targeting of depression. However, beyond these classical glial cell types, the functional heterogeneity and regulatory mechanisms of oligodendrocytes and their precursor cells within the neuroinflammatory microenvironment remain largely unexplored. Furthermore, expanding beyond a CNS-centric perspective to investigate the potential remote modulatory effects of peripheral organs (e.g., the gut) on CNS neuroinflammation may contribute to a more integrative understanding of systemic immune dysregulation in depression and offer novel theoretical perspectives for research and treatment.

#### 3.2.5 Microbial-gut-brain axis

The term MGB axis refers to a complex network of interconnected biological systems that enables bidirectional communication between gut microbiota and the brain. Maintaining homeostasis among the microbiota, CNS, and gastrointestinal tract is essential for overall health (67). Recent studies have revealed that individuals with MDD exhibit altered diversity and composition of gut microbiota, which strongly correlate with levels of inflammatory factors (68). Research into the relationship between gut microbiota and neuroimmune mechanisms shows that gut dysbiosis induces depression-like behavior via complement C3-mediated abnormalities in microglial synaptic pruning (69). Additionally, gut microbiota can modulate NLRP3 inflammasome involvement in the pathogenesis of depression. For example, CUMS can cause the dysbiosis of gut microbiota, increase harmful bacteria, disrupt the intestinal barrier, and permit bacterial metabolites such as LPS to enter the bloodstream. This activates NLRP3 inflammasomes in immune cells, exacerbates CNS inflammation, and triggers depression-like behavior (70). In addition, chronic ethanol exposure (CEE) induces gut microbiota dysbiosis and impairs gut homeostasis, resulting in elevated circulating LPS and inflammatory cytokines that activate hippocampal NLRP3 inflammasomes, resulting in neuroinflammation and depression-like symptoms (71). In NLRP3-deficient mice, gut microbiota regulates astrocyte dysfunction through circular RNA HIPK2, thereby ameliorating depression-like behavior (72).

The primary byproducts of microbial fermentation of dietary fiber are short-chain fatty acids (SCFAs), which are essential for maintaining immune function, neurological health, and metabolic balance (73). Shen et al. found that CEE leads to gut microbiota dysbiosis and reduces SCFA levels. This reduction damages gut structure and function, promotes CNS inflammation, disrupts the blood-brain barrier, causes neurological nutritional deficiencies, and leads to neuronal injury, ultimately resulting in anxiety- and depression-like behaviors. Importantly, fecal microbiota transplantation combined with SCFA supplementation significantly alleviates these adverse effects (74). In addition, in inflammatory bowel disease (IBD), perforin produced by colon CD8^+^ T cells promotes the expression of C-X-C motif chemokine ligand 9 (CXCL9), inducing ERS in hippocampal neurons and exacerbating depression associated with IBD (75). The splenic nerves may play a key role in LPS-induced depression-like phenotypes and inflammatory responses, while gut microbiota may regulate the function of microglia through the MGB axis, contributing to depression-like behaviors (76). Furthermore, mutations in the Fzd6 gene regulate the composition of gut microbiota by reducing the relative abundance of inflammation-related bacterial families such as *Ruminococcaceae* and *Lachnospiraceae*, which significantly mediates neuroinflammatory processes associated with depression (77).

In conclusion, the MGB axis plays a critical regulatory role in the onset and progression of depression through multiple mechanisms that alter the neuroimmune microenvironment, especially by mediating the aberrant activation of the NLRP3 inflammasome and microglia. Importantly, molecularly targeted interventions aimed at modulating the gut microbiota offer a crucial theoretical foundation for developing novel antidepressant therapies. Future research should integrate gut microbiome analysis, neuroimaging techniques, and molecular pharmacology to establish a multimodal research framework. This approach will systematically clarify the specific regulatory mechanisms of the MGB axis in depression and advance its potential as a therapeutic target.

#### 3.2.6 Programmed cell death

The development and homeostasis of multicellular organisms depend on regulating cell proliferation and the timely removal of harmful cells, such as damaged cells that may become cancerous or cells exploited by pathogens. This process is primarily achieved through PCD (78). PCD includes apoptosis, autophagy, and pyroptosis, all involving tightly regulated gene expression events (79). Pyroptosis is a lytic and inflammatory form of PCD, typically triggered by inflammasomes and executed by gasdermin (GSDM) proteins (80). In endocrine diseases, hyperglycemia induces apoptosis and pyroptosis of hippocampal neurons via an NLRP3-dependent pathway, which is associated with depression-like symptoms in STZ-induced diabetes models (81). In depression, deficiency of Kir6.1 activates the NLRP3 inflammasome through excessive accumulation of mitochondrial ROS, triggering caspase-1-dependent cleavage of gasdermin D (GSDMD), leading to astrocyte pyroptosis and the release of the pro-inflammatory cytokine IL-1β (82). Similarly, Li et al. found that CMS induces hippocampal astrocyte pyroptosis via activation of the NLRP3/caspase-1/GSDMD signaling pathway, causing a reduction in astrocyte numbers and depression-like behavior (83). In addition, knockout of the NLRP3 gene in astrocytes ameliorates depression-like pathology in mice with mild traumatic brain injury by inhibiting astrocytic pyroptosis (84). Methamphetamine exposure activates the NLRP6 inflammasome in astrocytes through regulation of miR-152, leading to caspase-1 cleavage, maturation and release of IL-1β and IL-18, and GSDMD pore formation on the plasma membrane. This triggers pyroptosis and results in pathological behaviors including depression (85). Moreover, ghrelin reduces neuroinflammation and alleviates depression-like behavior by inhibiting activation of the NLRP2/NLRP3 inflammasomes and pyroptosis in astrocytes (86).

Autophagy is a cellular process that enables the degradation and recycling of proteins and organelles to maintain cellular homeostasis (87). Dysfunctional autophagy worsens neuroinflammatory responses and induces depression-like behavior through the NLRP1-PI3K/Akt/mTOR signaling axis (88). The autophagy process is related to the activation of NLRP3 inflammasomes. Impaired lysosomal function within the autophagy-lysosome pathway can delay the degradation of NLRP3 inflammasomes, promoting the production of pro-inflammatory cytokines and leading to depressive behavior in CUMS mouse models (89). The high mobility group box 1/signal transducer and activator of transcription 3/p65 axis plays a key role in chronic stress-induced depression by driving microglial activation and autophagy (90). In addition, melatonin reduces autophagic damage by regulating FOXO3a, thereby preventing neuroinflammation and alleviating depressive symptoms (91). In summary, pyroptosis and autophagy-related regulatory mechanisms within PCD play significant roles in neuroinflammatory responses. Emerging research suggests their involvement in the pathological progression of depression and therapeutic outcomes. However, a systematic understanding of the specific molecular mechanisms underlying autophagy and pyroptosis in neuroinflammation, as well as their potential crosstalk, is currently lacking. There is an urgent need to deeply explore their intrinsic connections and synergistic interaction networks.

#### 3.2.7 Mitochondrion

Mitochondria are essential organelles found in most eukaryotic cells, responsible for key physiological processes such as energy production, signal transmission and regulation, maintenance of intracellular calcium balance, and ROS generation. When mitochondrial biogenesis is impaired, ROS levels increase, mitochondrial autophagy is impaired, and mitochondrial dynamics are altered, leading to mitochondrial dysfunction (92). Recent studies have shown that CUMS alters inflammation and brain mitochondrial activity, causing rats to exhibit depression-like behaviors. The mitochondrial function marker ATP is negatively correlated with levels of pro-inflammatory cytokines (93). This suggests a potential link between mitochondria and neuroinflammation in depression. Mechanistically, TNF-α inhibits NIP3-like protein X-mediated mitochondrial autophagy, leading to mitochondrial dysfunction and synaptic defects that trigger passive stress responses (94). Chronic restraint stress (CRS) increases cell-free mitochondrial DNA levels, activating the Toll-like receptor 9 signaling pathway, which induces neuroinflammation and social behavior deficits (95). Furthermore, mitochondrial uncoupling protein 2 (UCP2) regulates the ROS-thioredoxin-interacting protein (TXNIP)-NLRP3 signaling pathway to mediate NLRP3 inflammasome activation in astrocytes. UCP2 deficiency worsens CMS-induced depression-like behaviors, impairs neurogenesis, and causes astrocyte loss (96). O-[3-piperidino-2-hydroxy-1-propyl]-nicotinic acid amidoxime dihydrochloride (BGP-15) alleviates depression-like behavior by promoting PTEN-induced putative kinase 1/Parkin-dependent mitophagy, which mitigates LPS-induced mitochondrial dysfunction, neuroinflammation, and neuronal apoptosis (97).

#### 3.2.8 Neuroplasticity

Neuroplasticity is fundamental to brain development, learning, and CNS homeostasis (98). Neuroinflammation affects neuronal plasticity in the basolateral amygdala (BLA), leading to behaviors resembling anxiety and depression. For instance, LPS induces anxiety- and depression-like behaviors by activating microglia in the BLA, which enhances excitatory synaptic transmission and increases intrinsic neuronal excitability (99). In addition, chronic stress triggers inflammation induced by microglia and macrophages, worsening defects in synaptic phagocytosis and neuronal plasticity, leading to neurological dysfunction and depression-related behaviors (100). The transcription factor nuclear receptor subfamily 4 group A member 2 (Nr4a2), predominantly expressed in CNS neurons, plays a vital role in synaptic plasticity (101). Recent studies show that Nr4a2 can modulate depression-like behavior induced by LPS by reducing the morphological and functional damage caused by chronic neuroinflammation to microglia and calcium/calmodulin-dependent protein kinase II (CaMKII)-expressing neurons (102).

Downregulating mammalian STE20-like kinase 1 in the hippocampus significantly alleviates depression-like behavior and restores synaptic plasticity impaired by chronic stress by inhibiting the p38 signaling pathway and neuroinflammation (103). Targeting phosphatase and actin regulator 4 (Phactr4) also reverses chronic stress-induced depression-like behavior in rats by regulating neuroinflammation and neuroplasticity (104). Sleep deprivation reduces depression-like behavior in CRS mice by reducing neuroinflammatory responses in the ACC and improving neuroplasticity in both the PFC and ACC (105). Pharmacologically, activation of BDNF/tropomyosin receptor kinase B (TrkB) signaling improves synaptic plasticity and suppresses pro-inflammatory cytokine expression, resulting in rapid antidepressant-like effects (106). Leflunacetam ameliorates LPS-induced synaptic plasticity deficits and neuroinflammation by activating the BDNF/TrkB-mediated PI3K/Akt/mTOR signaling pathway (107). Additionally, pramipexole improves the depressive behavior in diabetic rats by inhibiting NLRP3 inflammasome-mediated neuroinflammation and protecting neural plasticity (108).

#### 3.2.9 Other types

Epigenetics is the study of heritable changes in gene expression caused by modifications that do not alter the DNA sequence itself (109). One key mechanism is DNA methylation, which regulates numerous cellular processes (110). In patients with MDD, five significant differential methylation positions were identified in the NLRP3 gene. The methylation levels at these sites correlated with abnormal changes in cortical thickness across the occipital, parietal, temporal, and frontal lobes, suggesting that NLRP3 inflammasome-associated neuroinflammation may mediate brain structural remodeling through epigenetic mechanisms (111). Moreover, immunomethylation patterns serve as a potential tool to stratify immune-related subtypes of MDD (112). Abnormal hydroxymethylation of the BDNF gene in the hippocampus, driven by neuroinflammation, is another important epigenetic factor associated with depression-like behavior (113). Furthermore, epigenetics-related circular RNAs may contribute to depression pathogenesis. For instance, Cai et al. found that circular RNA ubiquitin conjugating enzyme E2 K (circ-UBE2K) promotes aberrant microglial activation and neuroinflammation by binding to heterogeneous nuclear ribonucleoprotein U and regulating UBE2K expression, thus contributing to MDD development (114). Polycomb group 1 alleviates neuroinflammation and improves depressive behaviors by epigenetic inhibition of matrix metalloproteinase 10 (MMP10) expression in microglia (115).

**Figure 1:**
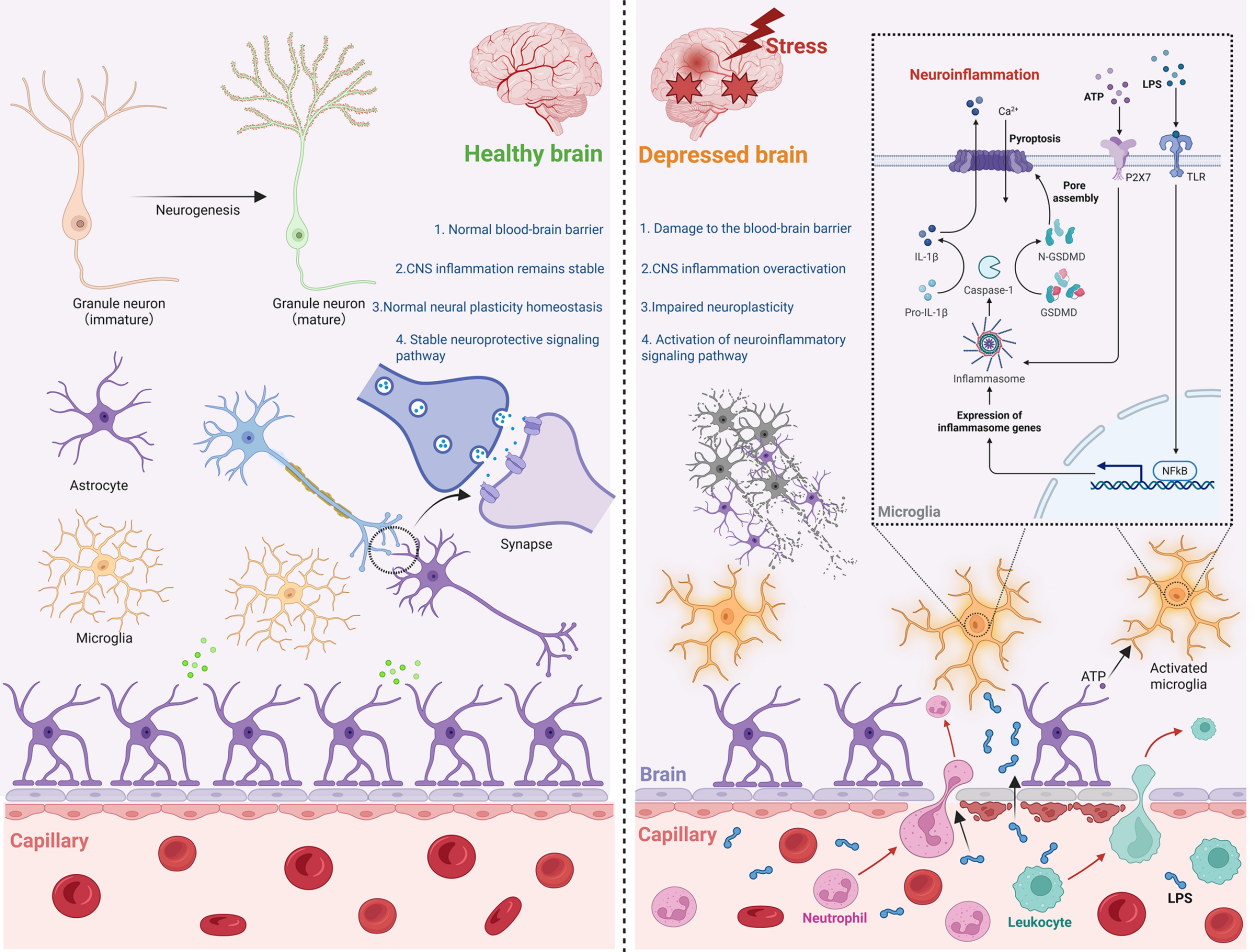
Neuroimmune mediated depression. In a healthy brain, the BBB remains structurally intact, neuronal functions are normal, neurotransmitter homeostasis is maintained, and glial cells show no signs of aberrant activation or damage. Conversely, in the context of depression under stress, BBB integrity is compromised. This breach allows peripheral harmful agents to enter the CNS, triggering aberrant microglial activation. Consequently, associated inflammatory signaling pathways become hyperactive, ultimately leading to neuronal injury. CNS, central nervous system. ATP, Adenosine Triphosphate. LPS, lipopolysaccharide. NF-κB, nuclear factor-kappaB. GSDM, gasdermin.

In living organisms, the metabolism of carbohydrates, lipids, proteins, and other substances follows a circadian rhythm to maintain the energy supply and material circulation necessary for normal biological functions. Circadian rhythms are crucial for organismal health but can be disrupted by pathological conditions, which may accelerate disease progression and create a vicious cycle (116). Brain and muscle ARNT-like protein 1 (BMAL1) is an important transcription factor that regulates these circadian rhythms (117). LPS triggers an “inflammatory storm” by activating microglia, disrupting the balance of circadian rhythm genes, particularly BMAL1, and promoting depression-like behavior by impairing synaptic plasticity and hypothalamic-pituitary-adrenal axis function (118). In addition, acute sleep deprivation in mice causes dysregulation of circadian rhythm-related gene expression and imbalances in gut microbiota, leading to excessive neuroinflammation and depression-like behaviors (119).Period genes 2 mediate the association between neuroinflammation and depressive behavior by affecting the expression of BMAL1 and its regulation of chemokine (C-C motif) ligand 5 (RANTES) (120). Exposure to polystyrene microplastics induces depression-like behavior in zebrafish by disrupting core circadian clock genes and triggering overactive neuroinflammation, marked by increased pro-inflammatory cytokines and microglial activation (121). In models with disrupted circadian rhythms, agomelatine has been shown to alleviate depression-like behavior, inhibit neuroinflammation, and promote hippocampal neurogenesis (122) (**Figure 1**).

In summary, research on the molecular mechanisms underlying neuroinflammation in depression reveals a complex pathological network involving coordinated interactions across multiple systems. Pro-inflammatory cytokines, inflammasome activation, and dysfunctional glial cells form the core pathological basis of neuroinflammation in depression. Key biological processes, such as mitochondrial dysfunction, disruption of the MGB axis, abnormal PCD, impaired neuroplasticity, epigenetic modifications, and circadian rhythm disturbances, contribute to the progression of depression by causing imbalances within neuroinflammatory networks, either directly or through cascading effects. Importantly, targeted modulation of these interconnected molecular pathways represents a promising direction for the development of developing novel antidepressant therapies.

Under this research framework, the multi-target regulatory strategy based on natural products demonstrates unique advantages. By systematically investigating how natural bioactive compounds modulate neuroinflammatory pathways, this approach not only deepens our understanding of the molecular pathology underlying depression but also identifies lead compounds with clearly defined therapeutic targets for clinical translation. This integrated research paradigm, combining molecular mechanism exploration with natural product-based drug development, holds promise for advancing antidepressant discovery from single-target interventions to innovative multi-system regulation. Such progress may provide a strong scientific foundation for overcoming current limitations in antidepressant therapy.

## 4 Molecular mechanism of natural product

### 4.1 Flavonoids

Flavonoids, as specialized plant secondary metabolites, are low-molecular-weight natural compounds known for their broad-spectrum bioactivities, including anticancer, antioxidant, and anti-inflammatory effects (123). Bavachin, a natural flavonoid extracted from Fructus Psoraleae, inhibits the NF-κB pathway by targeting protein kinase C delta. This action reduces neuroinflammation and oxidative stress, thereby improving the survival and function of diabetic neurons and ultimately alleviating diabetes-induced depressive behavior in mice (124). Schaftoside, found in traditional Chinese herbs such as Dendrobium nobile, exerts antidepressant effects by decreasing pro-inflammatory cytokines (IL-1β, IL-6, and TNF-α) in the serum and hippocampus of mice (125). Phloretin, a natural dihydrochalcone primarily isolated from apples, inhibits the NF-κB-C3 axis and microglia-mediated synaptic phagocytosis, providing neuroprotection in depression models (126). Quercetin, a flavonoid abundant in fruits and vegetables, suppresses neuroinflammation and alleviates LPS-induced depressive symptoms by modulating the NLRP3/NF-κB/inducible nitric oxide synthase (iNOS) signaling pathway in microglia (127). Astragolin is a flavonoid glycoside derived from *Astragalus sinicus* L. It effectively improves LPS-induced depressive-like behavior through maintenance of BBB integrity, inhibition of microglial activation, reduction of pro-inflammatory cytokines, and regulation of the receptor-interacting serine/threonine-protein kinase 1 (RIPK1)/RIPK3/mixed lineage kinase domain-like protein (MLKL) and mTOR/NF-κB inflammatory signaling pathways (128). Baicalin, extracted from dried roots of *Scutellaria*, has been shown through network pharmacology to regulate neuroinflammation, apoptosis, and oxidative stress, contributing to its antidepressant effects (129). In addition, baicalin can inhibit the activation of the glycogen synthase kinase-3 beta (GSK3β)/NF-κB/NLRP3 pathway, promoting neuronal maturation and protecting against neuronal damage, thereby alleviating CUMS-induced depression-like behavior (130).

Hyperoside is a flavonol glycoside found in various herbs, including *Artemisia capillaris* (131). It downregulates pro-inflammatory cytokine levels in the serum, intestinal tissue, and hippocampus, while also improving gut microbiota dysbiosis and SCFA concentrations caused by CRS (132). In addition, hyperoside alleviates microglial polarization and neuroinflammation through the thioredoxin-1/NLRP1/caspase-1 signaling pathway, thereby reducing depression-like behavior in CSDS mice (133). Seabuckthorn (*Hippophae rhamnoides* L.) is an excellent dietary source of flavonoids. Flavonoids derived from seabuckthorn can alleviate CUMS-induced gut microbiota disruption and downregulate inflammation-related factors (134). Kaempferol, a naturally occurring flavonoid present in many fruits and vegetables (such as onions, broccoli, strawberries, and grapes),and traditional Chinese medicines like Ginkgo biloba (135). It inhibits NLRP3 inflammasome activation and improves depression-like behavior by modulating microglial polarization and shifting the balance between PPARγ and signal transducer and activator of transcription 1 (STAT1) signaling pathways (136). Furthermore, Kaempferol-3-O-sophoroside not only improves depression-like behavior in mice but also promotes BDNF production in the hippocampus and induces autophagy to reduce NLRP3-mediated neuroinflammation (137).

Puerarin is the main bioactive compound extracted from Pueraria lobata (138). It can alleviate gut microbiota dysbiosis, inhibit the expression of pro-inflammatory cytokines and NF-κB in rats, and effectively treat depression (139). Puerarin generally exhibits low toxicity in both animals and humans. Among its adverse reactions, febrile responses are most common, followed by drug-induced dermatitis and hemolytic reactions. Although the incidence of hemolytic reactions combined with anaphylactic shock is extremely low, this potentially life-threatening complication poses a significant clinical risk for the use of puerarin (140). Therefore, further systematic evaluation of puerarin’s toxicity and potential side effects is recommended. Neohesperidin, a citrus-derived flavonoid, acts as a health-promoting phytochemical due to its diverse bioactivities and favorable safety profile. Its antidepressant effects are mediated through modulation of the NLRP3 inflammasome pathway (141). Luteolin, a flavonoid widely distributed in plants such as honeysuckle, is primarily found in fruits, vegetables, and medicinal herbs (142). It alleviates CRS-induced depressive behaviors by promoting PPARγ-dependent Arg-1^+^ microglial polarization, which suppresses neuroinflammation and reverses phagocytosis-driven synaptic pruning (143). Luteolin has demonstrated a favorable safety profile in clinical settings, with no significant adverse reactions reported in preclinical studies or human trials (144). However, its long-term safety and potential risks still need to be systematically validated through multi-center, large-sample clinical studies, especially regarding its use in populations with special physiological conditions. Establishing a comprehensive evidence-based medicine framework is necessary to address these concerns.

### 4.2 Terpenoids

Terpenes represent the largest class of plant-derived natural products, characterized by diverse chemical structures and a wide range of biological activities (145). 5-O-methylvisammioside, extracted from Saposhnikoviae Radix, inhibits NF-κB pathway activation and alleviates depression-like behavior by targeting SRC kinase (146). Carvacrol, a monoterpene found in the essential oils of aromatic plants such as Origanum vulgaris and Thymus vulgaris, reduces oxidative stress and decreases hippocampal IL-1β and TNF-α levels, thereby improving depression-like behavior induced by CUMS (147). Escin, a natural triterpenoid saponin from *Aesculus chinensis* (Suoluozi), alleviates CUMS-induced depression-like behaviors by modulating both the BDNF/TrkB/CREB and Toll-like receptor 4 (TLR4)/Myeloid differentiation factor 88 (MyD88)/NF-κB signaling pathways (148). Hyperibone J, a bioactive compound from *Hypericum bellum*, exerts antidepressant effects by adenosine kinase (ADK)-mediated suppression of P2X7R/TLR4-driven neuroinflammation in microglia (149). Total triterpenoids from *Rosa roxburghii*, primarily Kaji-ichigoside F1, demonstrate neuroprotective effects by activating the PPARγ/C-X3-C motif chemokine receptor 1 (CX3CR1)/Nrf2 pathway and inhibiting the NF-κB/NLRP3 signaling cascade to ameliorate LPS-induced depression-like behaviors (150). Yomogin, a sesquiterpenoid from *Artemisia iwayomogi*, mitigates LPS-induced depression by suppressing glial activation, pro-inflammatory cytokine expression, and mitogen-activated protein kinase (MAPK)-mediated neuroinflammation (151). Oridonin, a diterpenoid isolated from *Rabdosia rubescens*, exerts antidepressant effects by disrupting the interaction between NLRP3 and NEK7, thereby inhibiting neuroinflammation and autophagy impairment (152). Lycopene, a potent lipophilic antioxidant abundant in tomatoes, alleviates hippocampal microglial pyroptosis in chronic stress models by inhibiting the cathepsin B/NLRP3 pathway (153, 154).

Patchouli alcohol, the active constituent of *Pogostemon cablin*, exhibits anti-inflammatory and neuroprotective properties. It improves microglia-mediated neurogenesis impairment by inhibiting NLRP3 inflammasome activation, contributing to its antidepressant effects (155). Aucubin, an iridoid glycoside found in *Eucommia ulmoides*, has been shown by Liu et al. to alleviate depression-like behaviors in CUMS mice by targeting the GR/NF-κB/NLRP3 signaling pathway (156). Geniposide, an iridoid compound abundant in Gardenia jasminoides, mitigates inflammatory responses and abnormal glucose metabolism in depressed mice (157). However, due to its toxicity profile, geniposide dosage should be strictly controlled in clinical settings to balance efficacy and safety. Its pharmacokinetics vary with administration routes and disease models (158). Therefore, personalized dosing strategies based on metabolic and distribution parameters are recommended to optimize treatment and achieve precision medicine goals. Paeoniflorin, a water-soluble monoterpene glycoside and the main active ingredient of *Paeonia lactiflora* Pall (159), exerts antidepressant effects through multiple mechanisms. It reduces neuroinflammation by inhibiting microglia-induced caspase-11-dependent pyroptosis, suppresses NLRP3 inflammasome activation by promoting mitophagy, and modulates the SIRT1-NF-κB-NLRP3/pyroptosis axis to attenuate microglial activation (160–162). Acute toxicity of paeoniflorin is relatively low, with subacute and chronic toxicity studies showing minimal adverse effects. Experimental data indicate no significant genetic toxicity or mutagenicity (163). However, given the complexity of plant-derived natural products, long-term safety monitoring and in-depth drug interaction studies are essential to fully assess their safety profiles and potential clinical risks.

### 4.3 Phenolic

Phenolic natural products are a diverse class of organic compounds widely found in nature. These secondary metabolites occur in plants, microorganisms, and some animals, exhibiting a broad range of biological activities and therapeutic potential. For example, Rhodomyrtone, a natural phenolic compound extracted from *Rhodomyrtus tomentosa*, improves depression-like behavior by reducing TNF-α and TNF receptor 1 expression, inhibiting astrocyte activation, and decreasing neuronal apoptosis (164). Resveratrol, a naturally occurring polyphenol abundant in grapes (165), plays a significant role in treating inflammatory depression. Its antidepressant effect is closely linked to an anti-inflammatory pathway mediated by hippocampal type 2 bitter taste receptors (166). In addition, resveratrol can alleviate inflammation and anxiety-like depression caused by maternal-infant separation by activating the Sirt1/NF-κB pathway (167). However, resveratrol has low oral bioavailability, and high doses may pose risks of nephrotoxicity and liver damage (168). Therefore, future research should focus on optimizing pharmacokinetics, exploring the molecular mechanisms of toxicity, and developing advanced drug delivery systems. Polydatin, a resveratrol derivative from *Polygonum cuspidatum*, alleviates depression-like behavior induced by CUMS primarily by inhibiting neuroinflammation and oxidative stress via the NF-κB and Nrf2 pathways (169). Paeonol, a small-molecule polyphenol mainly extracted from the traditional Chinese medicine *Cortex Moutan*, reduces neuroinflammation by suppressing the hypoxia-inducible factor 1 alpha signaling pathway in microglia, thereby alleviating depression (170).

Punicalin, a polyphenol found in pomegranate fruits, inhibits the TLR4/NF-κB signaling pathway and alleviates LPS-induced pathological behaviors, including depression (171). Raspberries, globally known for their pleasant flavor, contain raspberry ketone, a phenolic compound that reduces LPS-induced depression-like behaviors in mice by inhibiting the TLR4/NF-κB pathway and modulating the MGB axis (172). Magnolol, a natural phenolic compound derived from *Magnolia officinalis*, alleviates CUMS-induced depression by suppressing pro-inflammatory M1 microglial polarization and promoting anti-inflammatory M2 polarization through the Nrf2/heme oxygenase-1 (HO-1)/NLRP3 signaling pathway (173). Gastrodin, the primary phenolic glycoside from *Gastrodia elata*, mitigates LPS-induced neuroinflammation by modulating the Arg-1^+^ microglial phenotype via regulation of Nrf2 (174). Licochalcone A, a phenolic compound found in licorice, reduces gliosis, regulates microglial polarization, improves synaptic plasticity, and prevents cognitive decline and depression-like behavior caused by LPS (175).

### 4.4 Saponin

Saponins are a class of amphiphilic compounds with diverse structures, widely distributed in many popular herbal plants (176). Akebia saponin D, a triterpenoid extracted from *Dipsacus aspe*r, preserves hippocampal neurogenesis by suppressing microglial inflammation through the PI3K/Akt signaling pathway, thereby improving depression-like behaviors and cognitive deficits in mice (177). Quinoa saponin shows potential as a natural dietary supplement for the treatment and prevention of anxiety and depression by regulating the MGB axis and inhibiting the activation of the TLR4/MyD88/NF-κB pathway (178). Saikosaponins, a group of triterpenoid saponins mainly derived from Radix Bupleuri (179), exert antidepressant effects primarily by reducing P2X7 expression in the brains of depressed mice, lowering central inflammation, and significantly inhibiting neuronal pyroptosis (180). Specifically, Saikosaponin A alleviates depressive symptoms by inhibiting hippocampal inflammation, increasing monoamine neurotransmitter levels, and modulating gut microbiota composition (181). Saikosaponin B2 also improves depression-like behavior by reducing microglial activation via the TLR4/NF-κB pathway, preventing ferroptosis, maintaining calcium homeostasis, and alleviating ERS (182). Saikosaponin C ameliorates CSDS-induced depression-like behaviors by inhibiting DNA methyltransferase 1, thereby decreasing IL-6 methylation and expression, while enhancing synaptic plasticity (183). Despite these neuroimmune regulatory effects, Saikosaponins carry potential risks of liver injury, and their pharmacological and toxicological effects are dose-dependent. High doses also pose a risk of nephrotoxicity (184). Therefore, future research should focus on defining the therapeutic window through systematic toxicology studies, establishing dosage guidelines balancing efficacy and safety, and elucidating the molecular mechanisms underlying liver and kidney toxicity, providing a theoretical basis for their rational clinical use.

Gypenosides from *Gynostemma pentaphyllum* ameliorate LPS-induced depressive behaviors through multitarget mechanisms, including normalizing systemic inflammation, modulating glial polarization, restoring hippocampal synaptic plasticity, and inhibiting PFC NLRP3 signaling to reinstate neurohomeostasis (185). Gypenosides-14 suppress pro-inflammatory cytokine expression, inhibit astrocyte activation, and attenuate hyperactivation of the NF-κB pathway (186). Ginsenosides are natural triterpenoid saponins obtained from different parts of ginseng, with diverse pharmacological activities such as anti-aging, immune regulation, and cognitive improvement (187). In antidepressant treatment, Ginsenoside Rb1 regulates mitochondrial autophagy and the NF-κB pathway, inhibits astrocyte pyroptosis, and maintains neural homeostasis by suppressing inflammation and enhancing synaptic plasticity (188). Ginsenoside Rc exerts antidepressant effects by modulating neuroinflammation, astrocyte-microglia crosstalk, and apoptosis pathways in the prefrontal cortex (189). Ginsenoside Re significantly reduces pro-inflammatory cytokine expression, inhibits microglial overactivation, and regulates BDNF signaling, thereby protecting neurons (190). Although certain ginsenosides have accumulated substantial scientific evidence supporting their safety and efficacy, their pharmacokinetic properties still require optimization. Current challenges include imbalanced or suboptimal solubility profiles (water versus lipid solubility) and metabolic instability, which limit their bioavailability in translational medical applications (191). To address these limitations, it is urgent to enhance their bioavailability and pharmacokinetic stability through formulation technology innovations, structural modifications, and the development of novel drug delivery systems.

### 4.5 Carbohydrates

*Morinda officinalis* is one of the four famous herbs in southern China (192). *Morinda officinalis* oligosaccharides (MOOs) have been used to treat mild and moderate depressive episodes. In preclinical studies, MOOs alleviate depression-like behaviors in PSD rats, mainly by regulating the NLRP3 inflammasome in microglia (193). In addition, MOOs inactivate the MyD88/PI3K pathway through E2F transcription factor 2 (E2F2), thereby reducing inflammation and depression-like behavior induced by CMS (194). In hypertension complicated with depression, astrocytes trigger neuroinflammatory responses due to mitochondrial damage, and MOOs upregulate Mitofusin 2 (Mfn2) expression, activating mitochondrial autophagy via the PI3K/Akt/mTOR pathway to clear damaged mitochondria in astrocytes (195). The antidepressant effects of *Eucommiae cortex* polysaccharides involve modulation of the MGB axis, including improvement of CUMS-induced gut dysbiosis by increasing Lactobacillaceae abundance and reducing LPS release, while simultaneously inhibiting microglia-mediated TLR4/NF-κB/MAPK signaling to attenuate neuroinflammation (196). Inulin, a soluble dietary fiber widely present in plants such as Jerusalem artichoke (197), protects the integrity of the intestinal barrier and blood-brain barrier, regulates TLR4/MyD88/NF-κB signaling to alleviate neuroinflammatory responses, and enhances CREB/BDNF signaling to promote neurogenesis and synaptic plasticity (198). Similarly, Rattan Pepper Polysaccharide (RPP) modulates the MGB axis by improving dextran sulfate sodium salt (DSS)-induced gut microbiota dysbiosis and SCFA disturbances, while restoring intestinal Th17/Treg homeostasis. In the brain, RPP mitigates neuroinflammation by suppressing the TLR4/NF-κB signaling pathway and enhances synaptic function through activation of CREB/BDNF signaling, thereby alleviating brain inflammation triggered by gut-derived inflammatory factors crossing the BBB and improving depression-like behaviors (199). In summary, current research indicates that natural saccharide compounds exert antidepressant effects by modulating the TLR4/NF-κB signaling pathway and MGB axis function to correct neuroinflammation-mediated immune imbalances. However, much remains to be explored in this field. Future studies should systematically clarify the biological functions and mechanisms of additional saccharide molecules in neuroimmune regulation.

### 4.6 Alkaloid

Alkaloids are chemical molecules characterized by cyclic structures containing one or more basic nitrogen atoms (200). Higenamine, one of the earliest discovered benzylisoquinoline alkaloids, is known for its cardiotonic effects and was originally isolated from the traditional Chinese medicine *Aconiti Lateralis Radix Praeparata* (201). Higenamine can alleviate the inflammatory response following chronic unpredictable stress in rats by downregulating pro-inflammatory cytokine levels and improving astrocyte gap junctions (202). Arecoline, a bioactive alkaloid extracted from Areca nuts, reduces pro-inflammatory markers such as IL-1β and LPS in serum and colon, while enhancing hippocampal neural plasticity through increased expression of BDNF and postsynaptic density protein-95 (203). Matrine, a quinolizidine alkaloid derived from the traditional Chinese herb *Sophora alopecuroides*, acts on the MGB axis by modulating gut microbiota and metabolites, restoring intestinal barrier integrity, and reducing intestinal inflammation. This leads to decreased levels of pro-inflammatory cytokines in peripheral circulation and brain regions, along with upregulation of BDNF expression in the brain (204). Tabersonine is a natural alkaloid isolated from the medicinal plant *Catharanthus roseus* (205). In preclinical mouse models, tabersonine effectively inhibits NLRP3 inflammasome-mediated signaling, downregulates pro-inflammatory cytokine expression, inhibits the activation of microglia, and significantly improves LPS-induced depression-like behaviors (206).

Berberine is the main bioactive component of *Rhizoma coptidis* (207). Yang et al. found that berberine restricts NLRP3 inflammasome activity by promoting the binding of tripartite motif (TRIM)-containing protein 65 to NLRP3, enhancing NLRP3 ubiquitination, and reducing functional damage to hippocampal neurons in CUMS mice (208). In addition, in corticosterone-induced depression-like behavior, berberine inhibits NLRP3 inflammasome activation to alleviate neuroinflammation and rescues neuronal degeneration by improving synaptic plasticity and neurogenesis (209). In summary, berberine exerts its antidepressant effects mainly through targeted modulation of the NLRP3 inflammasome. However, whether it produces synergistic effects through other neuroimmune regulatory pathways requires further validation by multifaceted studies. From a safety perspective, berberine demonstrates a favorable profile at conventional therapeutic doses, although some patients experience transient gastrointestinal reactions such as diarrhea or constipation (210). Nevertheless, future research should focus on establishing a comprehensive systematic toxicological assessment and further investigating the compound’s *in vivo* metabolism to provide a stronger scientific basis for its rational clinical use.

### 4.7 Other types

Betaine is abundant in various sources, such as sugar beet (211). Betaine protects nerves against Methamphetamine-induced nerve injury by suppressing the activation of the NLRP3 inflammasome pathway in the hippocampus, lowering IL-1β and TNF-α production, relieving pathogenic activation of microglia, and enhancing synaptic plasticity (212). In addition, betaine can significantly improve pain-related depression-like behavior induced by complete Freund’s adjuvant, specifically by regulating the phenotypic polarization of microglia and astrocytes, as well as modulating the expression of pro-inflammatory cytokines in the hippocampus (213). Schisandra has been a herbaceous plant resource with dual medicinal and edible functions since ancient times. It has multiple health and therapeutic effects (214). One of its potential natural compounds, Schisandrin B, downregulates the NF-κ B/TLR4/MyD88 signaling pathway and MAPK signaling pathway through miR-124, restoring M1/M2 balance and alleviating depressive symptoms (215).Schisandra chinensis Lignans can promote polarization of microglia towards the M2 phenotype, thereby exerting neuroprotective effects by activating the cannabinoid receptor type-2/STAT6 signaling pathway, and further exerting antidepressant effects (216). Arctiin is the main bioactive component of *Fructus arctii*. In depression, arctiin can bind to P2X7R to exert neuroprotective effects, thereby inhibiting the P2X7R/NLRP3 inflammasome signaling pathway (217). Yamogen is a natural compound with bioactive properties obtained from the Dioscorea species. It inhibits ERS and microglial activation, providing antidepressant benefits in an LPS-induced depression paradigm (218). Cajaninstilbene acid is a stilbene compound isolated from pigeon pea. It can alleviate depression like behavior in mice by inhibiting TLR4/NF-κ B mediated neuroinflammation and enhancing autophagy (219). Fucosterol is a plant sterol typically extracted from alginate. It has been confirmed that fuchosterol has anti-inflammatory activity. For example, in depression, fucosterol can regulate the activity of the MAPK/extracellular signal regulated kinase (ERK) 1/2 signaling pathway to inhibit microglial activation and neuroinflammation, thereby improving depression-like behaviors (220).

### 4.8 Herbal extracts

**Figure 2:**
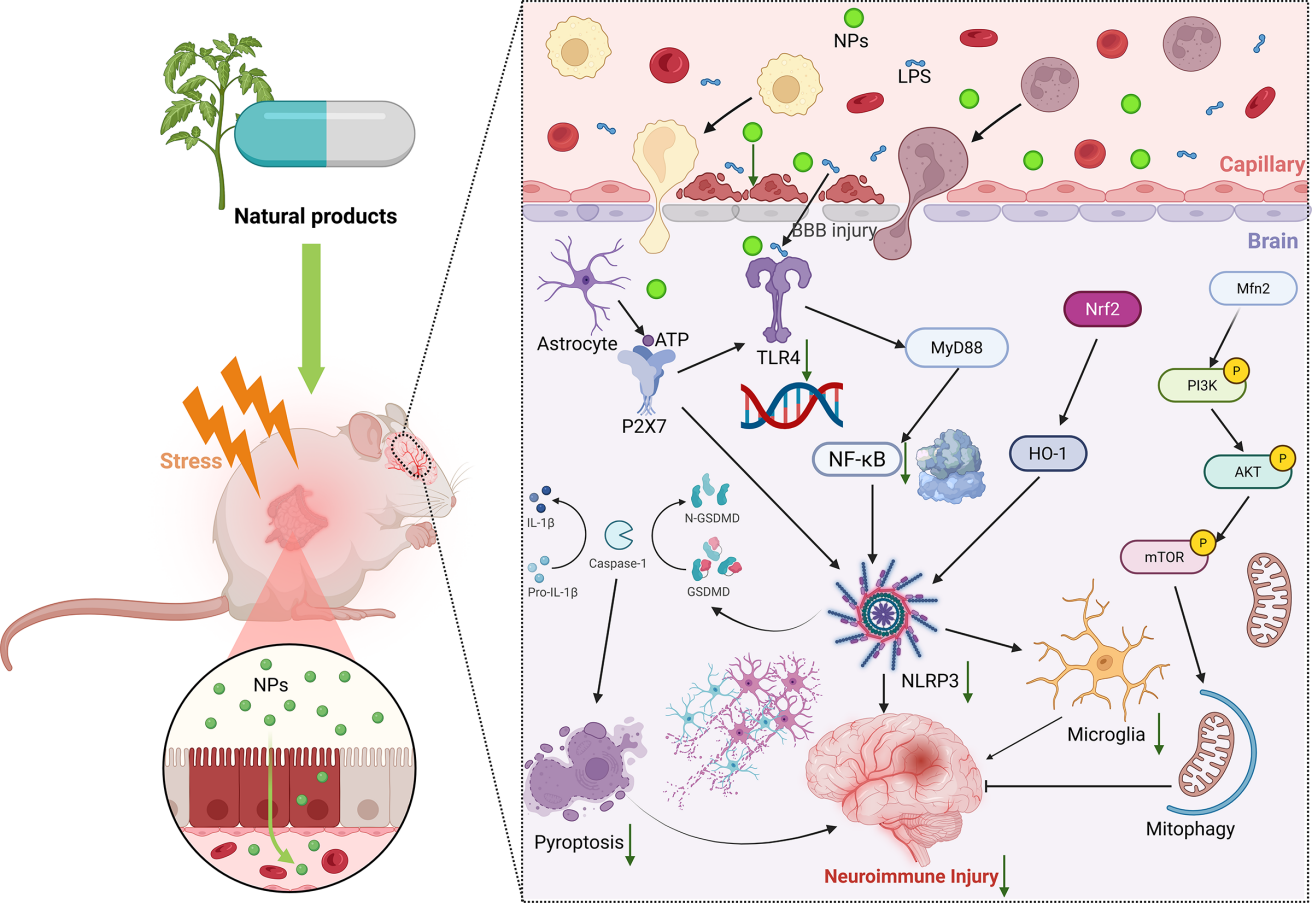
Natural products regulate neuroimmune molecular mechanisms. In the depressive state, administration of natural products can exert their multi-target and multi-pathway neuroimmunomodulatory properties, thereby mitigating neuronal injury. NPs, natural product. BBB, blood-brain barrier. ATP, Adenosine Triphosphate. LPS, lipopolysaccharide. NF-κB, nuclear factor-kappaB. GSDM, gasdermin. TLR4, toll-like receptor 4. NLRP3, NOD-like receptor family pyrin domain containing 3. Nrf2, nuclear factor E2-related factor 2. HO-1, heme oxygenase-1. Mfn2, Mitofusion 2. PI3K, phosphatidylinositol-3-kinase. AKT, protein kinase (B) mTOR, mammalian target of rapamycin.

Herbal extracts are natural and widely available active small-molecule ingredients commonly used to treat various diseases (221). *Withania somnifera* (L.) Dunal, known as Ashwagandha, is a small woody subshrub native to India, North Africa, and the Middle East. Its root extract suppresses the expression of inflammation-related proteins such as cyclooxygenase-2, iNOS, IL-6, IL-1β, and TNF-α, while modulating neuroendocrine and neurotransmitter systems to exert neuroprotective effects in mouse models of depression (222). *Cannabis sativa* L., an annual dioecious plant historically cultivated mainly in Central Asia, displays various pharmacological properties. Its inflorescence extract demonstrates antidepressant-like and anti-inflammatory effects in LPS-induced neuroinflammation models, potentially by regulating cytokine expression and mast cell activity (223). In addition, apple polyphenol extract inhibits MGB axis-mediated inflammatory responses, downregulates activation of the NF-κB inflammatory pathway, and significantly improves depression-like behavior (224). The methanolic extract of *Aerva javanica* leaves exerts antidepressant effects by downregulating brain tissue levels of TNF-α, IL-1β, and IL-6, inhibiting oxidative stress, and upregulating BDNF levels (225) (**Figure 2**; **Table 1**).

**Table 1:** Natural product research information.

Natural product	Main sources	PubChem CID	Molecular formula	Modeling method	Study dosage	Molecular mechanism	References
Bavachin	Fructus Psoraleae	14236566	C_20_H_20_O_4_	*In vivo*: Streptozotocin *In vitro*: LPS or high glucose	*In vivo*: 50 and 100 mg/kg *In vitro*: 5 and 10 μM	Acting on PKCδ to inhibit the NF- κB pathway	(124)
Schaftoside	Dendrobium nobile	442658	C_26_H_28_O_14_	CUMS and LPS	40, 80 and 160 mg/kg	Inhibit neuroinflammation	(125)
Phloretin	Apple	4788	C_15_H_14_O_5_	CMS	10, 20 and 50 mg/kg	Reduce synaptic phagocytosis mediated by microglia	(126)
Quercetin	Fruits and vegetables	5280343	C_15_H_10_O_7_	LPS	50 mg/kg	Inhibition of neuroinflammatory signaling pathway	(127)
Astragalin	*Astrolus sinicus* L	5282102	C_21_H_20_O_11_	LPS	*In vitro*: 0.2, 2 and 20 μM *In vivo*: 10 mg/kg	Maintaining blood-brain barrier integrity and inhibiting neuroinflammation	(128)
Baicalin	Scutellaria	64982	C_21_H_18_O_11_	CUMS	60 mg/kg	Inhibit neuroinflammation and cell apoptosis	(129)
				CUMS	20 and 40 mg/kg	Inhibition of GSK3 β/NF-κB/NLRP3 signaling pathway	(130)
Hyperoside	Artemisia capillaris	5281643	C_21_H_20_O_12_	CRS	9 and 36 mg/kg	Adjust MGB axis	(132)
				*In vivo*: CSDS *In vitro*: LPS	*In vivo*: 1.875 and 3.75 mg/kg *In vitro*: 5, 10 and 20 μM	Regulating the TRX1/NLRP1/Aspase-1 signaling pathway	(133)
Flavonoids from Seabuckthorn (*Hippophae rhamnoides* L.)	*Hippophae rhamnoides* L	–	–	CUMS	20 and 100 mg/kg	Adjust MGB axis	(134)
Kaempferol	*Ginkgo biloba*	C_15_H_10_O_6_	5280863	LPS	*In vivo*: 25 and 50 mg/kg *In vitro*: 5, 10, 20 and 40 μM	Balance of PPARγ and STAT1 signals	(136)
Kaempferol-3-O-sophoroside	*Crocus sativus* (Saffron)	C_27_H_30_O_16_	5282155	*In vivo*: CUMS *In vitro*: CORT	*In vivo*: 10 and 20 mg/kg *In vitro*: 12.5-50 μM	Binding with AMPK promotes the production of BDNF and enhances autophagy	(137)
Puerarin	Pueraria lobata	C_21_H_20_O_9_	5281807	CUMS	30 and 100 mg/kg	Adjust MGB axis	(139)
Neohesperidin	Citrus fruits	C_28_H_34_O_15_	442439	CUMS	25, 50 and 100 mg/kg	Regulating the NLRP3 signaling pathway	(141)
Luteolin	Honeysuckle	C_15_H_10_O_6_	5280445	CRS	10, 30 and 40 mg/kg	promoting the Arginase-1^+^ microglial phenotype	(143)
5-O-methylvisammioside	Saposhnikoviae Radix	C_22_H_28_O_10_	21670038	LPS	4 mg/kg	Inhibition of NF- κB pathway	(146)
Carvacrol	*Origanum vulgare* and *Thymus vulgaris*	C_10_H_14_O	10364	CUMS	50 mg/kg	Reduce oxidative stress and neuroinflammation	(147)
Escin	*Aesculus chinensis* (Suoluozi)	C_55_H_86_O_24_	16211024	CUMS	1, 3 and 10 mg/kg	Regulating BDNF/TrkB/CREB and TLR4/MyD88/NF-κB signaling pathways	(148)
Hyperibone J	*Hypericum bellum*	C_31_H_46_O_5_	44575718	*In vivo*: CUMS and LPS *In vitro*: LPS	*In vivo*: 10, 20 and 40 mg/kg *In vitro*: 10, 20 and 40 μM	Inhibition of neuroinflammation mediated by P2X7R/TLR4 in microglia	(149)
Kaji-ichigoside F1	*R. roxburghii*	C_36_H_58_O_10_	14019178	LPS	*In vivo*: 1, 2 and 4 mg/kg *In vitro*: 5, 10 and 20 μM	Inhibition of NF-κB/NLRP3 signaling pathway expression and activation of PPARγ/CX3CR1/Nrf2 signaling pathway	(150)
Yomogin	*Artemisia iwayomogi*	C_15_H_16_O_3_	174865	LPS	*In vivo*: 5 mg/kg *In vitro*: 0.1, 1 and 10 μM	Regulating the MAPK signaling pathway	(151)
Oridonin	*Rabdosia rubescens*	C_20_H_28_O_6_	5321010	*In vivo*: CUMS *In vitro*: LPS	*In vivo*: 5, 10 and 20 mg/kg *In vitro*: 5, 10, 20 and 40 μmol/L	Blocking the interaction between NLRP3 and NEK7 to inhibit neuroinflammation and autophagic damage	(152)
Lycopene	Tomato	C_40_H_56_	446925	CRS	50 mg/kg	Inhibition of cathepsin B/NLRP3 pathway	(154)
Patchouli alcohol	*Pogostemon cablin*	C_15_H_26_O	10955174	*In vivo*: CMS *In vitro*: LPS	*In vivo*: 10, 20 and 40 mg/kg *In vitro*: 5, 10, 20, 40 and 80 μM	Inhibition of NLRP3 inflammasome	(155)
Aucubin	*E.ulmoides*	C_15_H_22_O_9_	91458	*In vivo*: CUMS *In vitro*: CORT	*In vivo*: 10 and 20 mg/kg *In vitro*: 5, 10 and 20 μM	Regulating the GR/NF-κB/NLRP3 signaling pathway	(156)
Paeoniflorin	*Paeonia lactiflora pall*	C_23_H_28_O_11_	442534	*In vivo*: Reserpine *In vitro*: LPS and ATP	*In vivo*: 10, 20 and 40 mg/kg *In vitro*: 0, 1, 10 and 100 μM	Inhibition of CASP-11/GSDMD pathway	(160)
				*In vivo*: CUMS *In vitro*: CORT	*In vivo*: 20, 40 and 80 mg/kg *In vitro*: 50, 100 and 200 μmol/L	Promote mitochondrial autophagy and inhibit the activation of NLRP3 inflammasome	(161)
				*In vivo*: CUMS *In vitro*: LPS and ATP	*In vivo*: 40 and 80 mg/kg *In vitro*: 10 and 100 μM	Regulating SIRT1-NF-kB-NLRP3/pyroptosis pathway	(162)
Resveratrol	Grapes	C_14_H_12_O_3_	445154	CUMS	80 mg/kg	Inhibit neuroinflammation	(166)
				Maternal separation	40 mg/kg	Downregulation of Sirt1/NF-kB signaling pathway	(167)
Polydatin	*Polygonum cuspidatum*	C_20_H_22_O_8_	5281718	CUMS	100 and 200 mg/kg	Inhibited the activation of NF-κB and Nrf2 pathways	(169)
Paeonol	*Cortex Moutan*	C_9_H_10_O_3_	11092	LPS	*In vivo*: 10 and 20 mg/kg *In vitro*: 0, 5, 10, 20, 40, 60 and 80 μM	Regulating the HIF1A pathway	(170)
Punicalin	pomegranate fruits	C34H22O22	92131301	LPS	1, 5 and 10 μg/mL	Inhibition of TLR4/NF-κB pathway activity	(171)
Raspberry ketone	Raspberries	C_10_H_12_O_2_	21648	LPS	100, 200 and 400 mg/kg	Inhibition of TLR-4/NF-κB signaling pathway through MGB axis	(172)
Magnolol	*Magnolia officinalis*	C_18_H_18_O_2_	72300	*In vivo*: CUMS *In vitro*: LPS/ATP	*In vivo*: 50 and 100 mg/kg *In vitro*: 5, 10 and 20 μM	Regulating Nrf2/HO-1/NLRP3 signal transduction	(173)
Gastrodin	*Gastrodia elata*	C_13_H_18_O_7_	115067	LPS	25, 50 and 100 mg/kg	Promote Arg-1^+^microglial phenotype through Nrf2	(174)
Licochalcone A	Licorice	C_21_H_22_O_4_	5318998	LPS	15 mg/kg	Inhibit inflammation, enhance antioxidant response, prevent metabolic disorders, and improve synaptic related mechanisms	(175)
Akebia saponin D	Dipsacus asper Wall	C_47_H_76_O_18_	14284436	LPS	*In vivo*: 10, 50 and 100 mg/kg *In vitro*: 10, 50 and 100 μM	Activate PI3K-AKT pathway	(177)
Quinoa saponin	Quinoa	–	–	LPS	100 mg/kg	Inhibition of TLR4/MyD88/NF- κB pathway activation	(178)
Saikosaponins	Radix Bupleuri	–	–	LPS	120, 250 and 400 mg/kg	Inhibition of P2X7 receptor to alleviate neuronal pyroptosis	(180)
Saikosaponin A	Radix Bupleuri	C_42_H_68_O_13_	167928	Reserpine	50 mg/kg	Adjust MGB axis	(181)
Saikosaponins B2	Radix Bupleuri	C_42_H_68_O_13_	21637642	*In vivo*: CUMS *In vitro*: LPS	*In vivo*: 5 and 10 mg/kg *In vitro*: 0.2, 2 and 20 μM	Regulating the TLR4/NF-κB signaling pathway	(182)
Saikosaponin C	Radix Bupleuri	C_48_H_78_O_17_	167927	*In vivo*: CSDS *In vitro*: LPS	*In vivo*: 0.5 and 1 mg/kg *In vitro*: 0.5, 1 and 2 μM	Inhibition of DNMT1 protein reduces IL6 methylation	(183)
Gypenosides	*G. pentaphyllum*	–	–	LPS	50, 100 and 200 mg/kg	Inhibition of NLRP3/Caspase-1/ASC signaling pathway in PFC	(185)
Gypenosides-14	*G. pentaphyllum*	–	–	LPS	*In vivo*: 100 mg/kg *In vitro*: 30, 60 and 90 μM	Inhibition of NF- κB signaling pathway	(186)
Ginsenosides Rb1	Ginseng	C_54_H_92_O_23_	9898279	*In vivo*: CUMS *In vitro*: LPS	*In vivo*: 10 mg/kg *In vitro*: 0, 4, 8, 16, 32, and 64 µg/mL	Regulating mitochondrial autophagy and NF -κB pathway, inhibiting astrocyte pyroptosis	(188)
Ginsenosides Rc	Ginseng	C_53_H_90_O_22_	12855889	L-alpha-aminoadipic acid	20 mg/kg	Regulating neuroinflammation, astrocyte -microglial crosstalk, and apoptosis pathways	(189)
Ginsenosides Re	Ginseng	C_48_H_82_O_18_	441921	*In vivo*: Reserpine *In vitro*: H_2_O_2_	*In vivo*: 10, 30 and 90 mg/kg *In vitro*: 0.1, 2 and 10 μM	By inhibiting oxidative stress and inflammation, as well as activating the BDNF/TrkB/ERK/CREB pathway	(190)
*Morinda officinalis* oligosaccharides	*Morinda officinalis*	–	–	tMCAO and CUMS	0.1 mg/g	Regulating NLRP3 inflammasome in microglia	(193)
				*In vivo*: CMS *In vitro*: LPS	*In vivo*: 25 and 50 mg/kg *In vitro*: 2.5, 5, 10 mg/mL	Targeting the E2F2 mediated MyD88/PI3K signaling pathway	(194)
				*In vivo*: High-salt and CUMS *In vitro*: LPS	*In vivo*: 0.1 mg/g *In vitro*: 5 mg/mL	Upregulation of Mfn2 expression activates mitochondrial autophagy mediated by the PI3K/Akt/mTOR pathway	(195)
*Eucommiae cortex* polysaccharides	*Eucommiae cortex*	–	–	CUMS	100 mg/kg	Inhibiting gut microbiota mediated neuroinflammation	(196)
Inulin	Jerusalem artichoke	–	–	CUMS	0.037 g inulin/kcal	Regulating TLR4/MyD88/NF - κ B signaling transduction	(198)
Rattan Pepper Polysaccharide	Rattan Pepper	–	–	Dextran sulfate sodium salt	200 mg/kg	Adjust MGB axis	(199)
Higenamine	*Aconiti Lateralis Radix Praeparata*	C_16_H_17_NO_3_	114840	CUS	5, 10 and 20 mg/kg	Improve gap junctions and neuroinflammation in astrocytes	(202)
Arecoline	Areca nuts	C_8_H_13_NO_2_	2230	CUMS	10 mg/kg	Adjust MGB axis	(203)
Matrine	*S. alopecuroides*	C_15_H_24_N_2_O	91466	CUMS	15, 30 and 60 mg/kg	Adjust MGB axis	(204)
Tabersonine	*Catharanthus roseus*	C_21_H_24_N_2_O_2_	20485	*In vivo*: LPS *In vitro*: LPS and ATP	*In vivo*: 20 mg/kg *In vitro*: 1, 3, 6, 8, 10 and 20 μM	Inhibition of NLRP3 inflammasome activation	(206)
Berberine	*Rhizoma coptidis*	C_20_H_18_NO_4_^+^	2353	*In vivo*: CUMS *In vitro*: LPS and ATP	*In vivo*: 5 and 10 mg/kg *In vitro*: 0.5, 1, and 2 μM	Inhibit NLRP3 inflammasome	(208)
				CORT	100 and 200 mg/kg	Inhibition of NLRP3 inflammasome mediated neuroinflammation	(209)
Betaine	Sugar beet	C_5_H_11_NO_2_	247	Methamphetamine	10, 30 and 100 mg/kg	Inhibit NLRP3 inflammasome	(212)
				Complete Freund’s adjuvant	600 mg/kg	Changing the polarization of microglia and astrocytes to reduce neuroinflammation	(213)
Schisandrin B	*Schisandra*	C_23_H_28_O_6_	108130	LPS	31.25, 62.5, 125, 250, 500, and 1000 μg/mL	Downregulation of NF-κ B/TLR4/MyD88 signaling pathway	(215)
*Schisandra chinensis* Lignans	*Schisandra*	–	–	*In vivo*: CUMS *In vitro*: LPS	*In vivo*: 600 and 1200 mg/kg *In vitro*: 0−2200 μg/mL	Activate the CB2R/STAT6 signaling pathway	(216)
Arctiin	*Fructus arctii*	C_27_H_34_O_11_	100528	*In vivo*: CUMS *In vitro*: CORT	*In vivo*: 50 mg/kg *In vitro*: 0, 12.5, 25, 50, 100 and 200 μM	Inhibition of P2X7R/NLRP3 inflammasome signaling pathway	(217)
Yamogenin	Dioscorea species	C_27_H_42_O_3_	441900	LPS	5, 10 and 20 mg/kg	Inhibit endoplasmic reticulum stress and microglial activation	(218)
Cajaninstilbene acid	Pigeon pea	C_21_H_22_O_4_	9819225	CUMS and LPS	7.5, 15 and 30 mg/kg	Inhibition of TLR4/NF -κB mediated neuroinflammation and enhancement of autophagy	(219)
Fucosterol	Algae	C_29_H_48_O	5281328	CUMS and LPS	10 mg/kg	Inhibition of excessive activation of microglia by MAPK/ERK1/2 signaling inhibition	(220)
Ashwagandha root extract	Ashwagandha (*Withania somnifera* (L.) Dunal)	–	–	*In vivo*: CUMS *In vitro*: CORT	*In vivo*: 60 and 100 mg/kg *In vitro*: 100 and 200 μg/mL	Neuroprotection	(222)
Cannabis sativa L. inflorescence extract	*Cannabis sativa* L. inflorescence	–	–	LPS	10, 20 and 30 mg/kg	Regulating cytokine expression and mast cell activity	(223)
Apple polyphenol extract	Apple	–	–	High sucrose diet	500 mg/kg	Adjust MGB axis	(224)
Methanolic Extract of *Aerva javanica* Leaves	*Aerva javanica*	–	–	LPS	100, 300, and 500 mg/kg	Downregulate pro-inflammatory cytokines and inhibit oxidative stress	(225)

In summary, plant-derived natural products show great therapeutic potential for depression associated with neuroinflammation. Current evidence suggests that certain bioactive phytochemicals can improve depression-like behaviors by modulating neuroinflammatory responses. However, the complex multi-component composition of plant extracts makes it challenging to clarify the synergistic interactions among their active constituents. Furthermore, the physicochemical properties of solvents used during extraction can greatly affect the bioavailability and pharmacodynamic stability of the final products. Therefore, future research should systematically characterize the neuroimmunomodulatory effects of plant extracts. Establishing standardized research protocols will lay a theoretical foundation for developing novel antidepressant strategies to overcome the limitations of existing treatments.

## 5 Limitations and challenges

MDD is a highly prevalent chronic psychiatric condition worldwide, yet its underlying pathophysiological mechanisms remain incompletely understood. This knowledge gap severely impedes advances in developing targeted therapeutic strategies. Although conventional first-line antidepressants, such as fluoxetine, show moderate efficacy, their use is limited by single-target mechanisms and significant adverse effects. Recent research highlights the crucial role of neuroimmune regulatory networks in the pathogenesis, progression, and treatment outcomes of depression, presenting a promising direction for novel therapeutic approaches.

Natural products offer unique advantages in antidepressant therapy due to their multi-component synergistic mechanisms, which enable regulation of multiple targets within the neuroimmune system. This aligns well with the holistic regulatory principles of complex biological systems. Additionally, natural products possess bidirectional regulatory properties, capable of modulating both physiological functions and psychological states. This therapeutic approach of “harmonizing physiological and psychological states” closely matches the individualized treatment philosophy emphasized by precision medicine. Building on this foundation, scientifically integrating the multi-target benefits and precise actions of natural products to create a multidimensional synergistic framework, similar to “cocktail therapy”, will be pivotal for overcoming the limitations of single-target treatments and improving overall antidepressant effectiveness. This strategy represents a critical frontier in current translational medicine research.

Nevertheless, although natural products have demonstrated significant antidepressant potential in preclinical studies and suggest modulatory effects on the neuroimmune system, there remains a lack of high-quality evidence from large-scale, randomized, double-blind, placebo-controlled clinical trials. Therefore, their ability to produce clinically meaningful relief of depressive symptoms, deliver therapeutic benefits through neuroimmune modulation, and demonstrate strong safety profiles with long-term treatment sustainability requires rigorous validation through well-designed and methodologically robust clinical studies.

Special attention must be given to the risks of drug-drug interactions when combining natural products with conventional antidepressants. Clinically, co-administration can lead to pharmacokinetic interactions; for instance, concurrent use of miltirone and fluoxetine may cause toxic effects due to metabolic disturbances in individuals who are poor metabolizers of CYP2D6 or carry functional variants (226). Therefore, urgent research is needed to elucidate metabolic alterations during combination therapy and establish personalized medication guidelines for clinical practice.

Furthermore, the inherent physicochemical properties of certain natural products—such as inadequate chemical stability, low bioavailability, and limited BBB permeability—pose significant challenges to the targeted delivery of active compounds to the CNS. Meanwhile, natural resources from traditional medicinal systems (e.g., Chinese herbal medicine), face widespread issues including significant heterogeneity in cultivation conditions, lack of standardized processing methods, and substantial batch-to-batch variations in active components. The absence of standardized quality control systems not only limits their clinical application but also impedes rigorous investigation of their pharmacodynamic mechanisms.

On the other hand, although classical behavioral paradigms in rodents—such as the open field test, sucrose preference test, and forced swim test—allow relative quantification of depression-like behaviors, they fall short in capturing the complex psychological dimensions unique to human depression. Importantly, glial cells, including microglia and astrocytes, which are key mediators of neuroimmune regulation, play critical roles in the bioactivity of natural products. However, species differences in neurobiology between rodents and humans limit the ability of current models to accurately mimic the temporal progression of psychiatric symptoms. Moreover, existing models often fail to systematically simulate essential clinical features, such as the chronic nature of depression. These limitations in biological validity and clinical relevance inherently restrict the effective translation of preclinical findings into clinical practice.

Moreover, any biologically active substance on Earth can produce pharmacological effects and may cause non-specific off-target effects on normal tissues (227). Natural products are no exception in exerting antidepressant effects and carry potential toxicological risks and side effects. However, current research still lacks systematic evaluation of the possible adverse reactions and long-term risks associated with natural products, especially when multi-component synergistic interactions occur and their potential impacts on normal tissues. The absence of a comprehensive safety evaluation system has become a major barrier limiting the clinical translation of natural products.

## 6 Conclusion

In conclusion, future research must urgently prioritize establishing a comprehensive clinical translation framework for natural products. This includes systematically conducting high-quality, multi-center, large-sample randomized double-blind controlled trials and developing standardized efficacy evaluation systems to objectively assess their antidepressant effects in clinical settings. Meanwhile, research should focus on exploring combination therapy models that integrate natural products with conventional antidepressants. Using adaptive clinical trial designs, the clinical benefits of these combined regimens should be rigorously validated under strict safety and efficacy monitoring. Furthermore, close surveillance of patients’ pharmacokinetic profiles and safety parameters is essential to ensure robust protection during clinical application.

Additionally, overcoming the bottleneck of low CNS delivery efficiency for natural products is crucial. This urgently requires the development of novel, intelligent targeted delivery systems. For example, priority should be given to exploring advanced delivery strategies based on multifunctional nanocarriers (such as liposomes and polymeric nanoparticles) combined with BBB-penetrating technologies, including active targeting ligands or cell-penetrating peptides. Meanwhile, establishing a standardized, whole-process quality control system for herbal medicines is essential to provide a solid scientific foundation for the clinical translation of natural products. In addition, future research can focus on the neuroimmune pathways mediated by neural circuits, and further explore the molecular mechanisms of natural products such as quercetin, ginsenosides, and resveratrol that have a certain research foundation, laying a solid foundation for their clinical translation.

More importantly, future research must integrate cutting-edge technological platforms such as single-cell transcriptomics and spatial multi-omics to systematically elucidate the dynamic molecular mechanisms underlying neuroimmune pathology in depression. Based on these insights, a dynamic staging model of the chronic evolution of depression’s neuroimmune pathology should be developed to identify optimal time windows for intervention with natural products. Furthermore, humanized organoid models combined with targeted gene editing technologies can be employed to deeply explore core, evolutionarily conserved pathological mechanisms across species, thereby optimizing and validating personalized treatment strategies.

In summary, there is an urgent need for systematic exploration in both preclinical and clinical translational research focused on the neuroimmune mechanisms of depression and targeted intervention strategies. Such efforts will provide essential theoretical support and clear translational pathways for developing novel antidepressants (especially natural products), while significantly accelerating their clinical application.

## References

[1] Cui L Li S Wang S Wu X Liu Y Yu W. Major depressive disorder: hypothesis, mechanism, prevention and treatment. Signal Transduct Target Ther. (2024) 9:30. doi: 10.1038/s41392-024-01738-y, PMID: 38331979 PMC10853571

[2] Wang YS Shen CY Jiang JG. Antidepressant active ingredients from herbs and nutraceuticals used in TCM: pharmacological mechanisms and prospects for drug discovery. Pharmacol Res. (2019) 150:104520. doi: 10.1016/j.phrs.2019.104520, PMID: 31706012

[3] Wei Y Chang L Hashimoto K. Molecular mechanisms underlying the antidepressant actions of arketamine: beyond the NMDA receptor. Mol Psychiatry. (2022) 27:559–73. doi: 10.1038/s41380-021-01121-1, PMID: 33963284 PMC8960399

[4] Jiao W Lin J Deng Y Ji Y Liang C Wei S. The immunological perspective of major depressive disorder: unveiling the interactions between central and peripheral immune mechanisms. J Neuroinflammation. (2025) 22:10. doi: 10.1186/s12974-024-03312-3, PMID: 39828676 PMC11743025

[5] Bai Y Cai Y Chang D Li D Huo X Zhu T. Immunotherapy for depression: Recent insights and future targets. Pharmacol Ther. (2024) 257:108624. doi: 10.1016/j.pharmthera.2024.108624, PMID: 38442780

[6] Drevets WC Wittenberg GM Bullmore ET Manji HK. Immune targets for therapeutic development in depression: towards precision medicine. Nat Rev Drug Discov. (2022) 21:224–44. doi: 10.1038/s41573-021-00368-1, PMID: 35039676 PMC8763135

[7] Xing P Zhong Y Cui X Liu Z Wu X. Natural products in digestive tract tumors metabolism: Functional and application prospects. Pharmacol Res. (2023) 191:106766. doi: 10.1016/j.phrs.2023.106766, PMID: 37061144

[8] Fernandes A Rodrigues PM Pintado M Tavaria FK. A systematic review of natural products for skin applications: Targeting inflammation, wound healing, and photo-aging. Phytomedicine. (2023) 115:154824. doi: 10.1016/j.phymed.2023.154824, PMID: 37119762

[9] Pandey GN Zhang H Sharma A Ren X. Innate immunity receptors in depression and suicide: upregulated NOD-like receptors containing pyrin (NLRPs) and hyperactive inflammasomes in the postmortem brains of people who were depressed and died by suicide. J Psychiatry Neurosci. (2021) 46:E538–47. doi: 10.1503/jpn.210016, PMID: 34588173 PMC8526128

[10] Munshi S Alarbi AM Zheng H Kuplicki R Burrows K Figueroa-Hall L. Increased expression of ER stress, inflammasome activation, and mitochondrial biogenesis-related genes in peripheral blood mononuclear cells in major depressive disorder. Mol Psychiatry. (2025) 30:574–86. doi: 10.1038/s41380-024-02695-2, PMID: 39174649 PMC12054637

[11] Kang Y Shin D Kim A Tae WS Ham BJ Han KM. Resting-state functional connectivity is correlated with peripheral inflammatory markers in patients with major depressive disorder and healthy controls. J Affect Disord. (2025) 370:207–16. doi: 10.1016/j.jad.2024.11.017, PMID: 39521066

[12] Li C Ren H Liu H Li T Liu Y Wu B. Middle frontal gyrus volume mediates the relationship between interleukin-1β and antidepressant response in major depressive disorder. J Affect Disord. (2025) 372:56–65. doi: 10.1016/j.jad.2024.11.070, PMID: 39592061

[13] Gong W Zhai Q Wang Y Shen A Huang Y Shi K. Glymphatic function and choroid plexus volume is associated with systemic inflammation and oxidative stress in major depressive disorder. Brain Behav Immun. (2025) 128:266–275. doi: 10.1016/j.bbi.2025.04.008, PMID: 40220922

[14] Duan J Chen J Xiang Z. The U-shape relationship between the aggregate index of systemic inflammation and depression in American adults: A cross-sectional study. J Affect Disord. (2025) 380:270–8. doi: 10.1016/j.jad.2025.03.139, PMID: 40147607

[15] Jarkas DA Villeneuve AH Daneshmend AZB Villeneuve PJ McQuaid RJ. Sex differences in the inflammation-depression link: A systematic review and meta-analysis. Brain Behav Immun. (2024) 121:257–68. doi: 10.1016/j.bbi.2024.07.037, PMID: 39089535

[16] Edmondson-Stait AJ Davyson E Shen X Adams MJ Khandaker GM Miron VE. Associations between IL-6 and trajectories of depressive symptoms across the life course: Evidence from ALSPAC and UK Biobank cohorts. Eur Psychiatry. (2025) 68:e27. doi: 10.1192/j.eurpsy.2025.7, PMID: 39865800 PMC11883784

[17] Köhler CA Freitas TH Maes M de Andrade NQ Liu CS Fernandes BS. Peripheral cytokine and chemokine alterations in depression: a meta-analysis of 82 studies. Acta Psychiatr Scand. (2017) 135:373–87. doi: 10.1111/acps.12698, PMID: 28122130

[18] Cayrol C Girard JP. Interleukin-33 (IL-33): A nuclear cytokine from the IL-1 family. Immunol Rev. (2018) 281:154–68. doi: 10.1111/imr.12619, PMID: 29247993

[19] Li Y Gao W Jiao L Dong D Sun L Liu Y. Changes in mitochondrial transcriptional rhythms and depression-like behavior in the hippocampus of IL-33-overexpressing mice. Int J Mol Sci. (2025) 26:1895. doi: 10.3390/ijms26051895, PMID: 40076523 PMC11900197

[20] Xiao H Cao Y Lizano P Li M Sun H Zhou X. Interleukin-1β moderates the relationships between middle frontal-mACC/insular connectivity and depressive symptoms in bipolar II depression. Brain Behav Immun. (2024) 120:44–53. doi: 10.1016/j.bbi.2024.05.029, PMID: 38777282

[21] Li M Han L Xiao J Zhang S Liu G Sun X. IL-1ra treatment prevents chronic social defeat stress-induced depression-like behaviors and glutamatergic dysfunction via the upregulation of CREB-BDNF. J Affect Disord. (2023) 335:358–70. doi: 10.1016/j.jad.2023.05.049, PMID: 37217098

[22] Li M Li C Yu H Cai X Shen X Sun X. Lentivirus-mediated interleukin-1β (IL-1β) knock-down in the hippocampus alleviates lipopolysaccharide (LPS)-induced memory deficits and anxiety- and depression-like behaviors in mice. J Neuroinflammation. (2017) 14:190. doi: 10.1186/s12974-017-0964-9, PMID: 28931410 PMC5607621

[23] Wang Z. The intellectual base and research fronts of IL-18: A bibliometric review of the literature from WoSCC (2012-2022). Cell Prolif. (2024) 57:e13684. doi: 10.1111/cpr.13684, PMID: 39188114 PMC11533073

[24] Yamanishi K Doe N Mukai K Hashimoto T Gamachi N Hata M. Acute stress induces severe neural inflammation and overactivation of glucocorticoid signaling in interleukin-18-deficient mice. Transl Psychiatry. (2022) 12:404. doi: 10.1038/s41398-022-02175-7, PMID: 36151082 PMC9508168

[25] Ng SM Yin MXC Chan JSM Chan CHY Fong TCT Li A. Impact of mind-body intervention on proinflammatory cytokines interleukin 6 and 1β: A three-arm randomized controlled trial for persons with sleep disturbance and depression. Brain Behav Immun. (2022) 99:166–76. doi: 10.1016/j.bbi.2021.09.022, PMID: 34634445

[26] Ge J Li X Xia Y Chen Z Xie C Zhao Y. Recent advances in NLRP3 inflammasome in corneal diseases: Preclinical insights and therapeutic implications. Ocul Surf. (2024) 34:392–405. doi: 10.1016/j.jtos.2024.09.007, PMID: 39357820

[27] Zhang Y Liu L Liu YZ Shen XL Wu TY Zhang T. NLRP3 inflammasome mediates chronic mild stress-induced depression in mice via neuroinflammation. Int J Neuropsychopharmacol. (2015) 18:pyv006. doi: 10.1093/ijnp/pyv006, PMID: 25603858 PMC4571628

[28] Jeon SA Lee E Hwang I Han B Park S Son S. NLRP3 inflammasome contributes to lipopolysaccharide-induced depressive-like behaviors via indoleamine 2,3-dioxygenase induction. Int J Neuropsychopharmacol. (2017) 20:896–906. doi: 10.1093/ijnp/pyx065, PMID: 29016824 PMC5737528

[29] Li JM Hu T Zhou XN Zhang T Guo JH Wang MY. The involvement of NLRP3 inflammasome in CUMS-induced AD-like pathological changes and related cognitive decline in mice. J Neuroinflammation. (2023) 20:112. doi: 10.1186/s12974-023-02791-0, PMID: 37165444 PMC10173607

[30] Xu Y Sheng H Bao Q Wang Y Lu J Ni X. NLRP3 inflammasome activation mediates estrogen deficiency-induced depression- and anxiety-like behavior and hippocampal inflammation in mice. Brain Behav Immun. (2016) 56:175–86. doi: 10.1016/j.bbi.2016.02.022, PMID: 26928197

[31] Zhou M Tao X Lin K Leng C Yang Y Gui Y. Downregulation of the HCN1 channel alleviates anxiety- and depression-like behaviors in mice with cerebral ischemia-reperfusion injury by suppressing the NLRP3 inflammasome. J Am Heart Assoc. (2025) 14:e038263. doi: 10.1161/JAHA.124.038263, PMID: 40207529 PMC12132894

[32] Di Virgilio F Dal Ben D Sarti AC Giuliani AL Falzoni S. The P2X7 receptor in infection and inflammation. Immunity. (2017) 47:15–31. doi: 10.1016/j.immuni.2017.06.020, PMID: 28723547

[33] Pang F Yang Y Huang S Yang Z Zhu Z Liao D. Electroacupuncture alleviates depressive-like behavior by modulating the expression of P2X7/NLRP3/IL-1β of prefrontal cortex and liver in rats exposed to chronic unpredictable mild stress. Brain Sci. (2023) 13:436. doi: 10.3390/brainsci13030436, PMID: 36979246 PMC10046261

[34] Ren C Li LX Dong AQ Zhang YT Hu H Mao CJ. Depression induced by chronic unpredictable mild stress increases susceptibility to parkinson’s disease in mice via neuroinflammation mediated by P2X7 receptor. ACS Chem Neurosci. (2021) 12:1262–72. doi: 10.1021/acschemneuro.1c00095, PMID: 33734697

[35] Yue N Huang H Zhu X Han Q Wang Y Li B. Activation of P2X7 receptor and NLRP3 inflammasome assembly in hippocampal glial cells mediates chronic stress-induced depressive-like behaviors. J Neuroinflammation. (2017) 14:102. doi: 10.1186/s12974-017-0865-y, PMID: 28486969 PMC5424302

[36] Li Y Zhang Y Lin D Fu X Jing C. Demyelination of the amygdala mediates psychological stress-induced emotional disorders partially contributed by activation of P2X7R/NLRP3 cascade. Brain Behav Immun. (2025) 124:365–75. doi: 10.1016/j.bbi.2024.12.023, PMID: 39689840

[37] Li S Zhang Y Wang Y Zhang Z Xin C Wang Y. Transcutaneous vagus nerve stimulation modulates depression-like phenotype induced by high-fat diet via P2X7R/NLRP3/IL-1β in the prefrontal cortex. CNS Neurosci Ther. (2024) 30:e14755. doi: 10.1111/cns.14755, PMID: 38752512 PMC11097256

[38] Hao R Gao X Lu Q Zhao T Lu X Zhang F. CUMS induces depressive-like behaviors and cognition impairment by activating the ERS-NLRP3 signaling pathway in mice. J Affect Disord. (2025) 369:547–58. doi: 10.1016/j.jad.2024.10.001, PMID: 39378914

[39] Chen H Deng J Gao H Song Y Zhang Y Sun J. Involvement of the SIRT1-NLRP3 pathway in the inflammatory response. Cell Commun Signal. (2023) 21:185. doi: 10.1186/s12964-023-01177-2, PMID: 37507744 PMC10375653

[40] Abdel Rasheed NO Shiha NA Mohamed SS Ibrahim WW. SIRT1/PARP-1/NLRP3 cascade as a potential target for niacin neuroprotective effect in lipopolysaccharide-induced depressive-like behavior in mice. Int Immunopharmacol. (2023) 123:110720. doi: 10.1016/j.intimp.2023.110720, PMID: 37562290

[41] Arioz BI Tastan B Tarakcioglu E Tufekci KU Olcum M Ersoy N. Melatonin attenuates LPS-induced acute depressive-like behaviors and microglial NLRP3 inflammasome activation through the SIRT1/nrf2 pathway. Front Immunol. (2019) 10:1511. doi: 10.3389/fimmu.2019.01511, PMID: 31327964 PMC6615259

[42] Pallarés-Moratalla C Bergers G. The ins and outs of microglial cells in brain health and disease. Front Immunol. (2024) 15:1305087. doi: 10.3389/fimmu.2024.1305087, PMID: 38665919 PMC11043497

[43] Wu J Li Y Huang Y Liu L Zhang H Nagy C. Integrating spatial and single-nucleus transcriptomic data elucidates microglial-specific responses in female cynomolgus macaques with depressive-like behaviors. Nat Neurosci. (2023) 26:1352–64. doi: 10.1038/s41593-023-01379-4, PMID: 37443281

[44] Wu K Liu YY Shao S Song W Chen XH Dong YT. The microglial innate immune receptors TREM-1 and TREM-2 in the anterior cingulate cortex (ACC) drive visceral hypersensitivity and depressive-like behaviors following DSS-induced colitis. Brain Behav Immun. (2023) 112:96–117. doi: 10.1016/j.bbi.2023.06.003, PMID: 37286175

[45] Cao P Chen C Liu A Shan Q Zhu X Jia C. Early-life inflammation promotes depressive symptoms in adolescence via microglial engulfment of dendritic spines. Neuron. (2021) 109:2573–2589.e9. doi: 10.1016/j.neuron.2021.06.012, PMID: 34233151

[46] Brás JP Guillot de Suduiraut I Zanoletti O Monari S Meijer M Grosse J. Stress-induced depressive-like behavior in male rats is associated with microglial activation and inflammation dysregulation in the hippocampus in adulthood. Brain Behav Immun. (2022) 99:397–408. doi: 10.1016/j.bbi.2021.10.018, PMID: 34793941

[47] Naughton SX Yang EJ Iqbal U Trageser K Charytonowicz D Masieri S. Permethrin exposure primes neuroinflammatory stress response to drive depression-like behavior through microglial activation in a mouse model of Gulf War Illness. J Neuroinflammation. (2024) 21:222. doi: 10.1186/s12974-024-03215-3, PMID: 39272155 PMC11396632

[48] Feng X Zhao Y Yang T Song M Wang C Yao Y. Glucocorticoid-driven NLRP3 inflammasome activation in hippocampal microglia mediates chronic stress-induced depressive-like behaviors. Front Mol Neurosci. (2019) 12:210. doi: 10.3389/fnmol.2019.00210, PMID: 31555091 PMC6727781

[49] Pan Y Chen XY Zhang QY Kong LD. Microglial NLRP3 inflammasome activation mediates IL-1β-related inflammation in prefrontal cortex of depressive rats. Brain Behav Immun. (2014) 41:90–100. doi: 10.1016/j.bbi.2014.04.007, PMID: 24859041

[50] Su WJ Li JM Zhang T Cao ZY Hu T Zhong SY. Microglial NLRP3 inflammasome activation mediates diabetes-induced depression-like behavior via triggering neuroinflammation. Prog Neuropsychopharmacol Biol Psychiatry. (2023) 126:110796. doi: 10.1016/j.pnpbp.2023.110796, PMID: 37209992

[51] Tian Y Li M Zhang S Hu J Wu H Wan M. Microglia activation in the hippocampus mediates retinal degeneration-induced depressive-like behaviors via the NLRP3/IL-1β pathway. Brain Res Bull. (2023) 192:70–9. doi: 10.1016/j.brainresbull.2022.10.021, PMID: 36332880

[52] Yu X Yang T Wu D Xu C Li Z Sun A. PARP14 inhibits microglial activation via NNT to alleviate depressive-like behaviors in mice. Brain Behav Immun. (2025) 126:235–46. doi: 10.1016/j.bbi.2025.02.017, PMID: 39978699

[53] Hellmann-Regen J Clemens V Grözinger M Kornhuber J Reif A Prvulovic D. Effect of minocycline on depressive symptoms in patients with treatment-resistant depression: A randomized clinical trial. JAMA Netw Open. (2022) 5:e2230367. doi: 10.1001/jamanetworkopen.2022.30367, PMID: 36103181 PMC9475381

[54] Bassett B Subramaniyam S Fan Y Varney S Pan H Carneiro AMD. Minocycline alleviates depression-like symptoms by rescuing decrease in neurogenesis in dorsal hippocampus via blocking microglia activation/phagocytosis. Brain Behav Immun. (2021) 91:519–30. doi: 10.1016/j.bbi.2020.11.009, PMID: 33176182

[55] Wang R Ji L Yuan S Liu X Liang Z Chen W. Microglial forkhead box O3a deficiency attenuates LPS-induced neuro-inflammation and depressive-like behavior through regulating the expression of peroxisome proliferator-activated receptor-γ. Br J Pharmacol. (2024) 181:3908–25. doi: 10.1111/bph.16474, PMID: 38881194

[56] Lee HG Lee JH Flausino LE Quintana FJ. Neuroinflammation: An astrocyte perspective. Sci Transl Med. (2023) 15:eadi7828. doi: 10.1126/scitranslmed.adi7828, PMID: 37939162

[57] Xie XH Lai WT Xu SX Di Forti M Zhang JY Chen MM. Hyper-inflammation of astrocytes in patients of major depressive disorder: Evidence from serum astrocyte-derived extracellular vesicles. Brain Behav Immun. (2023) 109:51–62. doi: 10.1016/j.bbi.2022.12.014, PMID: 36587855

[58] Shen SY Liang LF Shi TL Shen ZQ Yin SY Zhang JR. Microglia-derived interleukin-6 triggers astrocyte apoptosis in the hippocampus and mediates depression-like behavior. Adv Sci (Weinh). (2025) 12:e2412556. doi: 10.1002/advs.202412556, PMID: 39888279 PMC11923973

[59] Wang JY Ren P Cui LY Duan JY Chen HL Zeng ZR. Astrocyte-specific activation of sigma-1 receptors in mPFC mediates the faster onset antidepressant effect by inhibiting NF-κB-induced neuroinflammation. Brain Behav Immun. (2024) 120:256–74. doi: 10.1016/j.bbi.2024.06.008, PMID: 38852761

[60] Guo Q Gobbo D Zhao N Zhang H Awuku NO Liu Q. Adenosine triggers early astrocyte reactivity that provokes microglial responses and drives the pathogenesis of sepsis-associated encephalopathy in mice. Nat Commun. (2024) 15:6340. doi: 10.1038/s41467-024-50466-y, PMID: 39068155 PMC11283516

[61] Novakovic MM Korshunov KS Grant RA Martin ME Valencia HA Budinger GRS. Astrocyte reactivity and inflammation-induced depression-like behaviors are regulated by Orai1 calcium channels. Nat Commun. (2023) 14:5500. doi: 10.1038/s41467-023-40968-6, PMID: 37679321 PMC10485021

[62] Gao R Ali T Liu Z Li A He K Yang C. NMDAR (2C) deletion in astrocytes relieved LPS-induced neuroinflammation and depression. Int Immunopharmacol. (2024) 132:111964. doi: 10.1016/j.intimp.2024.111964, PMID: 38603856

[63] Song Z Bian Z Zhang Z Wang X Zhu A Zhu G. Astrocytic Kir4.1 regulates NMDAR/calpain signaling axis in lipopolysaccharide-induced depression-like behaviors in mice. Toxicol Appl Pharmacol. (2021) 429:115711. doi: 10.1016/j.taap.2021.115711, PMID: 34474083

[64] Leng L Zhuang K Liu Z Huang C Gao Y Chen G. Menin deficiency leads to depressive-like behaviors in mice by modulating astrocyte-mediated neuroinflammation. Neuron. (2018) 100:551–563.e7. doi: 10.1016/j.neuron.2018.08.031, PMID: 30220511

[65] Liu Y Song N Yao H Jiang S Wang Y Zheng Y. β-Arrestin2-biased Drd2 agonist UNC9995 alleviates astrocyte inflammatory injury via interaction between β-arrestin2 and STAT3 in mouse model of depression. J Neuroinflammation. (2022) 19:240. doi: 10.1186/s12974-022-02597-6, PMID: 36183107 PMC9526944

[66] Wang X Su M Wang L Zhou Y Li N Yang B. NEDD4 like E3 ubiquitin protein ligase represses astrocyte activation and aggravates neuroinflammation in mice with depression via paired box 6 ubiquitination. Neuroscience. (2023) 530:144–57. doi: 10.1016/j.neuroscience.2023.08.036, PMID: 37661017

[67] Morais LH Schreiber HL4 Mazmanian SK. The gut microbiota-brain axis in behavior and brain disorders. Nat Rev Microbiol. (2021) 19:241–55. doi: 10.1038/s41579-020-00460-0, PMID: 33093662

[68] Zhang Q Zhao W Yun Y Ma T An H Fan N. Multiomics analysis reveals aberrant tryptophan-kynurenine metabolism and immunity linked gut microbiota with cognitive impairment in major depressive disorder. J Affect Disord. (2025) 373:273–83. doi: 10.1016/j.jad.2024.12.070, PMID: 39716675

[69] Hao W Ma Q Wang L Yuan N Gan H He L. Gut dysbiosis induces the development of depression-like behavior through abnormal synapse pruning in microglia-mediated by complement C3. Microbiome. (2024) 12:34. doi: 10.1186/s40168-024-01756-6, PMID: 38378622 PMC10877840

[70] Huang L Ma Z Ze X Zhao X Zhang M Lv X. Gut microbiota decreased inflammation induced by chronic unpredictable mild stress through affecting NLRP3 inflammasome. Front Cell Infect Microbiol. (2023) 13:1189008. doi: 10.3389/fcimb.2023.1189008, PMID: 37293210 PMC10244772

[71] Yao H Zhang D Yu H Yuan H Shen H Lan X. Gut microbiota regulates chronic ethanol exposure-induced depressive-like behavior through hippocampal NLRP3-mediated neuroinflammation. Mol Psychiatry. (2023) 28:919–30. doi: 10.1038/s41380-022-01841-y, PMID: 36280756 PMC9908543

[72] Zhang Y Huang R Cheng M Wang L. Chao J Li J Zheng P. Gut microbiota from NLRP3-deficient mice ameliorates depressive-like behaviors by regulating astrocyte dysfunction via circHIPK2. Microbiome. (2019) 7:116. doi: 10.1186/s40168-019-0733-3, PMID: 31439031 PMC6706943

[73] Ikeda T Nishida A Yamano M Kimura I. Short-chain fatty acid receptors and gut microbiota as therapeutic targets in metabolic, immune, and neurological diseases. Pharmacol Ther. (2022) 239:108273. doi: 10.1016/j.pharmthera.2022.108273, PMID: 36057320

[74] Shen H Zhang C Zhang Q Lv Q Liu H Yuan H. Gut microbiota modulates depressive-like behaviors induced by chronic ethanol exposure through short-chain fatty acids. J Neuroinflammation. (2024) 21:290. doi: 10.1186/s12974-024-03282-6, PMID: 39508236 PMC11539449

[75] Huang S Pan L Pang S Guo H Li M Tian Y. Perforin generated by CD8+ T cells exacerbates inflammatory bowel disease-induced depression by promoting CXCL9 production in intestinal epithelial cells. Gastroenterology. (2025) 169(2):294–307. doi: 10.1053/j.gastro.2025.02.036, PMID: 40120774

[76] Ma L Zhang J Fujita Y Shinno-Hashimoto H Shan J Wan X. Effects of spleen nerve denervation on depression-like phenotype, systemic inflammation, and abnormal composition of gut microbiota in mice after administration of lipopolysaccharide: A role of brain-spleen axis. J Affect Disord. (2022) 317:156–65. doi: 10.1016/j.jad.2022.08.087, PMID: 36037991

[77] Chen W Yan X Song X Yang Y Wang X Xu G. Effects of Fzd6 on intestinal flora and neuroinflammation in lipopolysaccharide-induced depression-like mice. J Affect Disord. (2025) 372:160–72. doi: 10.1016/j.jad.2024.12.011, PMID: 39643213

[78] Bedoui S Herold MJ Strasser A. Emerging connectivity of programmed cell death pathways and its physiological implications. Nat Rev Mol Cell Biol. (2020) 21:678–95. doi: 10.1038/s41580-020-0270-8, PMID: 32873928

[79] Li Z Li D Chen R Gao S Xu Z Li N. Cell death regulation: A new way for natural products to treat osteoporosis. Pharmacol Res. (2023) 187:106635. doi: 10.1016/j.phrs.2022.106635, PMID: 36581167

[80] Rao Z Zhu Y Yang P Chen Z Xia Y Qiao C. Pyroptosis in inflammatory diseases and cancer. Theranostics. (2022) 12:4310–29. doi: 10.7150/thno.71086, PMID: 35673561 PMC9169370

[81] Li DX Wang CN Wang Y Ye CL Jiang L Zhu XY. NLRP3 inflammasome-dependent pyroptosis and apoptosis in hippocampus neurons mediates depressive-like behavior in diabetic mice. Behav Brain Res. (2020) 391:112684. doi: 10.1016/j.bbr.2020.112684, PMID: 32454054

[82] Li F Jiang SY Tian T Li WJ Xue Y Du RH. Kir6.1/K-ATP channel in astrocytes is an essential negative modulator of astrocytic pyroptosis in mouse model of depression. Theranostics. (2022) 12:6611–25. doi: 10.7150/thno.77455, PMID: 36185602 PMC9516231

[83] Li S Sun Y Song M Song Y Fang Y Zhang Q. NLRP3/caspase-1/GSDMD-mediated pyroptosis exerts a crucial role in astrocyte pathological injury in mouse model of depression. JCI Insight. (2021) 6:e146852. doi: 10.1172/jci.insight.146852, PMID: 34877938 PMC8675200

[84] Miao HT Wang J Shao JJ Song RX Li WG Sun JK. Astrocytic NLRP3 cKO mitigates depression-like behaviors induced by mild TBI in mice. Neurobiol Dis. (2025) 205:106785. doi: 10.1016/j.nbd.2024.106785, PMID: 39793767

[85] Oladapo A Kannan M Deshetty UM Singh S Buch S Periyasamy P. Methamphetamine-mediated astrocytic pyroptosis and neuroinflammation involves miR-152-NLRP6 inflammasome signaling axis. Redox Biol. (2025) 80:103517. doi: 10.1016/j.redox.2025.103517, PMID: 39879739 PMC11810843

[86] Han X Fu X Guo W Liu Y Sun J Wang T. Ghrelin inhibits inflammasomes activation in astrocytes, alleviates pyroptosis, and prevents lipopolysaccharide-induced depression-like behavior in mice. Inflammation. (2024) 48(4):2292–312. doi: 10.1007/s10753-024-02190-4, PMID: 39702621 PMC12336091

[87] Liu S Yao S Yang H Liu S Wang Y. Autophagy: Regulator of cell death. Cell Death Dis. (2023) 14:648. doi: 10.1038/s41419-023-06154-8, PMID: 37794028 PMC10551038

[88] Zhu YJ Huang J Chen R Zhang Y He X Duan WX. Autophagy dysfunction contributes to NLRP1 inflammasome-linked depressive-like behaviors in mice. J Neuroinflammation. (2024) 21:6. doi: 10.1186/s12974-023-02995-4, PMID: 38178196 PMC10765763

[89] Li MM Wang X Chen XD Yang HL Xu HS Zhou P. Lysosomal dysfunction is associated with NLRP3 inflammasome activation in chronic unpredictable mild stress-induced depressive mice. Behav Brain Res. (2022) 432:113987. doi: 10.1016/j.bbr.2022.113987, PMID: 35780959

[90] Xu K Wang M Wang H Zhao S Tu D Gong X. HMGB1/STAT3/p65 axis drives microglial activation and autophagy exert a crucial role in chronic Stress-Induced major depressive disorder. J Adv Res. (2024) 59:79–96. doi: 10.1016/j.jare.2023.06.003, PMID: 37321346 PMC11081938

[91] Ali T Rahman SU Hao Q Li W Liu Z Ali Shah F. Melatonin prevents neuroinflammation and relieves depression by attenuating autophagy impairment through FOXO3a regulation. J Pineal Res. (2020) 69:e12667. doi: 10.1111/jpi.12667, PMID: 32375205

[92] Gu YY Zhao XR Zhang N Yang Y Yi Y Shao QH. Mitochondrial dysfunction as a therapeutic strategy for neurodegenerative diseases: Current insights and future directions. Ageing Res Rev. (2024) 102:102577. doi: 10.1016/j.arr.2024.102577, PMID: 39528070

[93] Wang L Xu Y Jiang M Wang M Ji M Xie X. Chronic stress induces depression-like behavior in rats through affecting brain mitochondrial function and inflammation. Psychoneuroendocrinology. (2025) 172:107261. doi: 10.1016/j.psyneuen.2024.107261, PMID: 39721083

[94] Lu JJ Wu PF He JG Li YK Long LH Yao XP. BNIP3L/NIX-mediated mitophagy alleviates passive stress-coping behaviors induced by tumor necrosis factor-α. Mol Psychiatry. (2023) 28:5062–76. doi: 10.1038/s41380-023-02008-z, PMID: 36914810

[95] Tripathi A Bartosh A Whitehead C Pillai A. Activation of cell-free mtDNA-TLR9 signaling mediates chronic stress-induced social behavior deficits. Mol Psychiatry. (2023) 28:3806–15. doi: 10.1038/s41380-023-02189-7, PMID: 37528226 PMC10730412

[96] Du RH Wu FF Lu M Shu XD Ding JH Wu G. Uncoupling protein 2 modulation of the NLRP3 inflammasome in astrocytes and its implications in depression. Redox Biol. (2016) 9:178–87. doi: 10.1016/j.redox.2016.08.006, PMID: 27566281 PMC5007434

[97] Liu Q Zhao JN Fang ZT Wang X Zhang BG He Y. BGP-15 alleviates LPS-induced depression-like behavior by promoting mitophagy. Brain Behav Immun. (2024) 119:648–64. doi: 10.1016/j.bbi.2024.04.036, PMID: 38677623

[98] Dzyubenko E Hermann DM. Role of glia and extracellular matrix in controlling neuroplasticity in the central nervous system. Semin Immunopathol. (2023) 45:377–87. doi: 10.1007/s00281-023-00989-1, PMID: 37052711 PMC10279577

[99] Zheng ZH Tu JL Li XH Hua Q Liu WZ Liu Y. Neuroinflammation induces anxiety- and depressive-like behavior by modulating neuronal plasticity in the basolateral amygdala. Brain Behav Immun. (2021) 91:505–18. doi: 10.1016/j.bbi.2020.11.007, PMID: 33161163

[100] Kokkosis AG Madeira MM Hage Z Valais K Koliatsis D Resutov E. Chronic psychosocial stress triggers microglial-/macrophage-induced inflammatory responses leading to neuronal dysfunction and depressive-related behavior. Glia. (2024) 72:111–32. doi: 10.1002/glia.24464, PMID: 37675659 PMC10842267

[101] Català-Solsona J Miñano-Molina AJ Rodríguez-Álvarez J. Nr4a2 transcription factor in hippocampal synaptic plasticity, memory and cognitive dysfunction: A perspective review. Front Mol Neurosci. (2021) 14:786226. doi: 10.3389/fnmol.2021.786226, PMID: 34880728 PMC8645690

[102] He Y Wang Y Yu H Tian Y Chen X Chen C. Protective effect of Nr4a2 (Nurr1) against LPS-induced depressive-like behaviors via regulating activity of microglia and CamkII neurons in anterior cingulate cortex. Pharmacol Res. (2023) 191:106717. doi: 10.1016/j.phrs.2023.106717, PMID: 36948326

[103] Yan Y Xu X Chen R Wu S Yang Z Wang H. Down-regulation of MST1 in hippocampus protects against stress-induced depression-like behaviors and synaptic plasticity impairments. Brain Behav Immun. (2021) 94:196–209. doi: 10.1016/j.bbi.2021.02.007, PMID: 33607238

[104] Li Y Chen X Lan T Wang W Wang C Chang M. Targeting Phactr4 to rescue chronic stress-induced depression-like behavior in rats via regulating neuroinflammation and neuroplasticity. Int J Biol Macromol. (2024) 273:132854. doi: 10.1016/j.ijbiomac.2024.132854, PMID: 38838879

[105] Shi S Zhang M Xie W Ju P Chen N Wang F. Sleep deprivation alleviates depression-like behaviors in mice via inhibiting immune and inflammatory pathways and improving neuroplasticity. J Affect Disord. (2023) 340:100–12. doi: 10.1016/j.jad.2023.07.119, PMID: 37543111

[106] Sun SR Zhao JN Bi PW Zhang HY Li GX Yan JZ. Pharmacologically activating BDNF/TrkB signaling exerted rapid-acting antidepressant-like effects through improving synaptic plasticity and neuroinflammation. Metab Brain Dis. (2025) 40:158. doi: 10.1007/s11011-025-01583-0, PMID: 40131536

[107] Wu Y Zhu Z Lan T Li S Li Y Wang C. Levomilnacipran improves lipopolysaccharide-induced dysregulation of synaptic plasticity and depression-like behaviors via activating BDNF/trkB mediated PI3K/akt/mTOR signaling pathway. Mol Neurobiol. (2024) 61:4102–15. doi: 10.1007/s12035-023-03832-8, PMID: 38057644

[108] Li P Wang T Guo H Liu Y Zhao H Ren T. Pramipexole improves depression-like behavior in diabetes mellitus with depression rats by inhibiting NLRP3 inflammasome-mediated neuroinflammation and preventing impaired neuroplasticity. J Affect Disord. (2024) 356:586–96. doi: 10.1016/j.jad.2024.04.073, PMID: 38657764

[109] Tsou PS Varga J O’Reilly S. Advances in epigenetics in systemic sclerosis: molecular mechanisms and therapeutic potential. Nat Rev Rheumatol. (2021) 17:596–607. doi: 10.1038/s41584-021-00683-2, PMID: 34480165

[110] Meng H Cao Y Qin J Song X Zhang Q Shi Y. DNA methylation, its mediators and genome integrity. Int J Biol Sci. (2015) 11:604–17. doi: 10.7150/ijbs.11218, PMID: 25892967 PMC4400391

[111] Han KM Choi KW Kim A Kang W Kang Y Tae WS. Association of DNA methylation of the NLRP3 gene with changes in cortical thickness in major depressive disorder. Int J Mol Sci. (2022) 23:5768. doi: 10.3390/ijms23105768, PMID: 35628578 PMC9143533

[112] Van Assche E Hohoff C Su Atil E Wissing SM Serretti A Fabbri C. Exploring the use of immunomethylomics in the characterization of depressed patients: A proof-of-concept study. Brain Behav Immun. (2025) 123:597–605. doi: 10.1016/j.bbi.2024.09.026, PMID: 39341467

[113] Zhao T Piao LH Li DP Xu SH Wang SY Yuan HB. BDNF gene hydroxymethylation in hippocampus related to neuroinflammation-induced depression-like behaviors in mice. J Affect Disord. (2023) 323:723–30. doi: 10.1016/j.jad.2022.12.035, PMID: 36529411

[114] Cai Y Ji Y Liu Y Zhang D Gong Z Li L. Microglial circ-UBE2K exacerbates depression by regulating parental gene UBE2K via targeting HNRNPU. Theranostics. (2024) 14:4058–75. doi: 10.7150/thno.96890, PMID: 38994030 PMC11234284

[115] Li N Du J Yang Y Zhao T Wu D Peng F. Microglial PCGF1 alleviates neuroinflammation associated depressive behavior in adolescent mice. Mol Psychiatry. (2025) 30:914–26. doi: 10.1038/s41380-024-02714-2, PMID: 39215186 PMC11835731

[116] Xin M Bi F Wang C Huang Y Xu Y Liang S. The circadian rhythm: A new target of natural products that can protect against diseases of the metabolic system, cardiovascular system, and nervous system. J Adv Res. (2025) 69:495–514. doi: 10.1016/j.jare.2024.04.005, PMID: 38631431 PMC11954810

[117] Zhang N Yu H Liu T Zhou Z Feng B Wang Y. Bmal1 downregulation leads to diabetic cardiomyopathy by promoting Bcl2/IP3R-mediated mitochondrial Ca2+ overload. Redox Biol. (2023) 64:102788. doi: 10.1016/j.redox.2023.102788, PMID: 37356134 PMC10320280

[118] Xu DD Hou ZQ Xu YY Liang J Gao YJ Zhang C. Potential role of bmal1 in lipopolysaccharide-induced depression-like behavior and its associated “Inflammatory storm. J Neuroimmune Pharmacol. (2024) 19:4. doi: 10.1007/s11481-024-10103-3, PMID: 38305948

[119] Yang DF Huang WC Wu CW Huang CY Yang YSH Tung YT. Acute sleep deprivation exacerbates systemic inflammation and psychiatry disorders through gut microbiota dysbiosis and disruption of circadian rhythms. Microbiol Res. (2023) 268:127292. doi: 10.1016/j.micres.2022.127292, PMID: 36608535

[120] Chen X Hu Q Zhang K Teng H Li M Li D. The clock-controlled chemokine contributes to neuroinflammation-induced depression. FASEB J. (2020) 34:8357–66. doi: 10.1096/fj.201900581RRR, PMID: 32329129

[121] Yang B Han Y Hu S Xie X Zhu X Yuan L. Polystyrene microplastics induce depression-like behavior in zebrafish via neuroinflammation and circadian rhythm disruption. Sci Total Environ. (2025) 959:178085. doi: 10.1016/j.scitotenv.2024.178085, PMID: 39708463

[122] Atanasova D Lazarov N Stoyanov DS Spassov RH Tonchev AB Tchekalarova J. Reduced neuroinflammation and enhanced neurogenesis following chronic agomelatine treatment in rats undergoing chronic constant light. Neuropharmacology. (2021) 197:108706. doi: 10.1016/j.neuropharm.2021.108706, PMID: 34274352

[123] Sun Q Liu Q Zhou X Wang X Li H Zhang W. Flavonoids regulate tumor-associated macrophages - From structure-activity relationship to clinical potential (Review). Pharmacol Res. (2022) 184:106419. doi: 10.1016/j.phrs.2022.106419, PMID: 36041653

[124] Zhang Z Sun L Guo Y Zhao J Li J Pan X. Bavachin ameliorates neuroinflammation and depressive-like behaviors in streptozotocin-induced diabetic mice through the inhibition of PKCδ. Free Radic Biol Med. (2024) 213:52–64. doi: 10.1016/j.freeradbiomed.2024.01.010, PMID: 38215890

[125] Hu Y Gan Y Lei J Cai J Zhou Y Chen H. Schaftoside reduces depression- and anxiogenic-like behaviors in mice depression models. Brain Sci. (2025) 15:238. doi: 10.3390/brainsci15030238, PMID: 40149760 PMC11940525

[126] Li C Liu B Xu J Jing B Guo L Wang L. Phloretin decreases microglia-mediated synaptic engulfment to prevent chronic mild stress-induced depression-like behaviors in the mPFC. Theranostics. (2023) 13:955–72. doi: 10.7150/thno.76553, PMID: 36793870 PMC9925308

[127] Adeoluwa OA Olayinka JN Adeoluwa GO Akinluyi ET Adeniyi FR Fafure A. Quercetin abrogates lipopolysaccharide-induced depressive-like symptoms by inhibiting neuroinflammation via microglial NLRP3/NFκB/iNOS signaling pathway. Behav Brain Res. (2023) 450:114503. doi: 10.1016/j.bbr.2023.114503, PMID: 37209878

[128] Cao MM Guo Z Wang J Ma HY Qin XY Hu Y. Astragalin alleviates lipopolysaccharide-induced depressive-like behavior in mice by preserving blood-brain barrier integrity and suppressing neuroinflammation. Free Radic Biol Med. (2025) 232:340–52. doi: 10.1016/j.freeradbiomed.2025.03.014, PMID: 40089077

[129] Yi Y Liu G Li Y Wang C Zhang B Lou H. Baicalin ameliorates depression-like behaviors via inhibiting neuroinflammation and apoptosis in mice. Int J Mol Sci. (2024) 25:10259. doi: 10.3390/ijms251910259, PMID: 39408591 PMC11476789

[130] Zhang CY Zeng MJ Zhou LP Li YQ Zhao F Shang ZY. Baicalin exerts neuroprotective effects via inhibiting activation of GSK3β/NF-κB/NLRP3 signal pathway in a rat model of depression. Int Immunopharmacol. (2018) 64:175–82. doi: 10.1016/j.intimp.2018.09.001, PMID: 30195108

[131] Wang S Sheng F Zou L Xiao J Li P. Hyperoside attenuates non-alcoholic fatty liver disease in rats via cholesterol metabolism and bile acid metabolism. J Adv Res. (2021) 34:109–22. doi: 10.1016/j.jare.2021.06.001, PMID: 35024184 PMC8655136

[132] Song A Cheng R Jiang J Qu H Wu Z Qian F. Antidepressant-like effects of hyperoside on chronic stress-induced depressive-like behaviors in mice: Gut microbiota and short-chain fatty acids. J Affect Disord. (2024) 354:356–67. doi: 10.1016/j.jad.2024.03.017, PMID: 38492650

[133] Zhao K Zhou F Lu Y Gao T Wang R Xie M. Hyperoside alleviates depressive-like behavior in social defeat mice by mediating microglial polarization and neuroinflammation via TRX1/NLRP1/Caspase-1 signal pathway. Int Immunopharmacol. (2025) 145:113731. doi: 10.1016/j.intimp.2024.113731, PMID: 39647288

[134] Xia CX Gao AX Zhu Y Dong TT Tsim KW. Flavonoids from Seabuckthorn (Hippophae rhamnoides L.) restore CUMS-induced depressive disorder and regulate the gut microbiota in mice. Food Funct. (2023) 14:7426–38. doi: 10.1039/d3fo01332d, PMID: 37485660

[135] Chen M Xiao J El-Seedi HR Woźniak KS Daglia M Little PJ. Kaempferol and atherosclerosis: From mechanism to medicine. Crit Rev Food Sci Nutr. (2024) 64:2157–75. doi: 10.1080/10408398.2022.2121261, PMID: 36099317

[136] Su P Liu L Gong Y Peng S Yan X Bai M. Kaempferol improves depression-like behaviors through shifting microglia polarization and suppressing NLRP3 via tilting the balance of PPARγ and STAT1 signaling. Int Immunopharmacol. (2024) 143:113538. doi: 10.1016/j.intimp.2024.113538, PMID: 39492132

[137] Wang R Hu X Liu S Wang J Xiong F Zhang X. Kaempferol-3-O-sophoroside (PCS-1) contributes to modulation of depressive-like behavior in C57BL/6J mice by activating AMPK. Br J Pharmacol. (2024) 181:1182–202. doi: 10.1111/bph.16283, PMID: 37949672

[138] Lin SP Zhu L Shi H Ye S Li Q Yin X. Puerarin prevents sepsis-associated encephalopathy by regulating the AKT1 pathway in microglia. Phytomedicine. (2023) 121:155119. doi: 10.1016/j.phymed.2023.155119, PMID: 37801894

[139] Song X Wang W Ding S Wang Y Ye L Chen X. Exploring the potential antidepressant mechanisms of puerarin: Anti-inflammatory response via the gut-brain axis. J Affect Disord. (2022) 310:459–71. doi: 10.1016/j.jad.2022.05.044, PMID: 35568321

[140] Meng F Guo B Ma YQ Li KW Niu FJ. Puerarin: A review of its mechanisms of action and clinical studies in ophthalmology. Phytomedicine. (2022) 107:154465. doi: 10.1016/j.phymed.2022.154465, PMID: 36166943

[141] Luo L Liu W Dong L Wang S Wang Q Jiang Y. Neohesperidin improves depressive-like behavior induced by chronic unpredictable mild stress in mice. Neurochem Res. (2025) 50:69. doi: 10.1007/s11064-024-04323-5, PMID: 39751909

[142] Kou JJ Shi JZ He YY Hao JJ Zhang HY Luo DM. Luteolin alleviates cognitive impairment in Alzheimer’s disease mouse model via inhibiting endoplasmic reticulum stress-dependent neuroinflammation. Acta Pharmacol Sin. (2022) 43:840–9. doi: 10.1038/s41401-021-00702-8, PMID: 34267346 PMC8975883

[143] Yuan NJ Zhu WJ Ma QY Huang MY Huo RR She KJ. Luteolin ameliorates chronic stress-induced depressive-like behaviors in mice by promoting the Arginase-1+ microglial phenotype via a PPARγ-dependent mechanism. Acta Pharmacol Sin. (2025) 46:575–91. doi: 10.1038/s41401-024-01402-9, PMID: 39496862 PMC11845711

[144] Vajdi M Karimi A Karimi M Abbasalizad Farhangi M Askari G. Effects of luteolin on sepsis: A comprehensive systematic review. Phytomedicine. (2023) 113:154734. doi: 10.1016/j.phymed.2023.154734, PMID: 36898254

[145] Zeng T Chen Y Jian Y Zhang F Wu R. Chemotaxonomic investigation of plant terpenoids with an established database (TeroMOL). New Phytol. (2022) 235:662–73. doi: 10.1111/nph.18133, PMID: 35377469

[146] Zhu W Li B Cui R. 5-O-Methylvisammioside alleviates depression-like behaviors by inhibiting nuclear factor kappa B pathway activation via targeting SRC. Neural Regener Res. (2025) 10.4103/NRR.NRR-D-24-00714. doi: 10.4103/NRR.NRR-D-24-00714, PMID: 40313111 PMC13452683

[147] Valentim JT da Silva DMA Capibaribe VCC Sales ISL Rebouças MO. Carvacrol alleviates CUMS-induced depressive-like behaviors and cognitive impairment by reducing oxidative stress and neuroinflammation in mice. Behav Brain Res. (2024) 472:115135. doi: 10.1016/j.bbr.2024.115135, PMID: 38964616

[148] Liu F Jia Y Zhao L Xiao LN Cheng X Xiao Y. Escin ameliorates CUMS-induced depressive-like behavior via BDNF/TrkB/CREB and TLR4/MyD88/NF-κB signaling pathways in rats. Eur J Pharmacol. (2024) 984:177063. doi: 10.1016/j.ejphar.2024.177063, PMID: 39426465

[149] Li T Li Y Chen J Nan M Zhou X Yang L. Hyperibone J exerts antidepressant effects by targeting ADK to inhibit microglial P2X7R/TLR4-mediated neuroinflammation. J Adv Res. (2025) 72:571–89. doi: 10.1016/j.jare.2024.07.015, PMID: 39019111 PMC12147645

[150] Huang M Chen F Zhou L Zhang Q Wang L Li L. The antidepressant effects of kaji-ichigoside F1 via activating PPAR-γ/CX3CR1/Nrf2 signaling and suppressing NF-κB/NLRP3 signaling pathways. Front Pharmacol. (2025) 16:1569888. doi: 10.3389/fphar.2025.1569888, PMID: 40308754 PMC12040888

[151] Kim JH Ju IG Kim N Huh E Son SR Hong JP. Yomogin, Isolated from Artemisia iwayomogi, Inhibits Neuroinflammation Stimulated by Lipopolysaccharide via Regulating MAPK Pathway. Antioxidants (Basel). (2022) 12:106. doi: 10.3390/antiox12010106, PMID: 36670968 PMC9854746

[152] Liang L Wang H Hu Y Bian H Xiao L Wang G. Oridonin relieves depressive-like behaviors by inhibiting neuroinflammation and autophagy impairment in rats subjected to chronic unpredictable mild stress. Phytother Res. (2022) 36:3335–51. doi: 10.1002/ptr.7518, PMID: 35686337

[153] Li N Wu X Zhuang W Xia L Chen Y Wu C. Tomato and lycopene and multiple health outcomes: Umbrella review. Food Chem. (2021) 343:128396. doi: 10.1016/j.foodchem.2020.128396, PMID: 33131949

[154] Zhu Q Gao Z Peng J Liu C Wang X Li S. Lycopene alleviates chronic stress-induced hippocampal microglial pyroptosis by inhibiting the cathepsin B/NLRP3 signaling pathway. J Agric Food Chem. (2023) 71:20034–46. doi: 10.1021/acs.jafc.3c02749, PMID: 38054647

[155] He H Xie X Zhang J Mo L Kang X Zhang Y. Patchouli alcohol ameliorates depression-like behaviors through inhibiting NLRP3-mediated neuroinflammation in male stress-exposed mice. J Affect Disord. (2023) 326:120–31. doi: 10.1016/j.jad.2023.01.065, PMID: 36682696

[156] Liu P Song S Yang P Rao X Wang Y Bai X. Aucubin improves chronic unpredictable mild stress-induced depressive behavior in mice via the GR/NF-κB/NLRP3 axis. Int Immunopharmacol. (2023) 123:110677. doi: 10.1016/j.intimp.2023.110677, PMID: 37523973

[157] Chen G Zhang W Chen Q Dong M Liu M Liu G. Geniposide exerts the antidepressant effect by affecting inflammation and glucose metabolism in a mouse model of depression. Chem Biol Interact. (2024) 400:111182. doi: 10.1016/j.cbi.2024.111182, PMID: 39098740

[158] Liu L Wu Q Chen Y Gu G Gao R Peng B. Updated pharmacological effects, molecular mechanisms, and therapeutic potential of natural product geniposide. Molecules. (2022) 27:3319. doi: 10.3390/molecules27103319, PMID: 35630796 PMC9144884

[159] Zhi SM Cui Y Liu Y Zhang JT Li XJ Sheng B. Paeoniflorin suppresses ferroptosis after traumatic brain injury by antagonizing P53 acetylation. Phytomedicine. (2024) 133:155940. doi: 10.1016/j.phymed.2024.155940, PMID: 39128303

[160] Tian DD Wang M Liu A Gao MR Qiu C Yu W. Antidepressant effect of paeoniflorin is through inhibiting pyroptosis CASP-11/GSDMD pathway. Mol Neurobiol. (2021) 58:761–76. doi: 10.1007/s12035-020-02144-5, PMID: 33025508

[161] Su L Guo P Guo X He Z Zhao Y Zong Y. Paeoniflorin alleviates depression by inhibiting the activation of NLRP3 inflammasome via promoting mitochondrial autophagy. Chin J Nat Med. (2024) 22:515–29. doi: 10.1016/S1875-5364(24)60654-0, PMID: 38906599

[162] Wang X Su L Liu S He Z Li J Zong Y. Paeoniflorin inhibits the activation of microglia and alleviates depressive behavior by regulating SIRT1-NF-kB-NLRP3/pyroptosis pathway. Int J Mol Sci. (2024) 25:12543. doi: 10.3390/ijms252312543, PMID: 39684254 PMC11640976

[163] Ou X Yu Z Pan C Zheng X Li D Qiao Z. Paeoniflorin: a review of its pharmacology, pharmacokinetics and toxicity in diabetes. Front Pharmacol. (2025) 16:1551368. doi: 10.3389/fphar.2025.1551368, PMID: 40260393 PMC12009869

[164] Gao M Song Y Liu Y Miao Y Guo Y Chai H. TNF-α/TNFR1 activated astrocytes exacerbate depression-like behavior in CUMS mice. Cell Death Discov. (2024) 10:220. doi: 10.1038/s41420-024-01987-4, PMID: 38710713 PMC11074147

[165] Liu N Fan X Shao Y Chen S Wang T Yao T. Resveratrol attenuates inflammation and fibrosis in rheumatoid arthritis-associated interstitial lung disease via the AKT/TMEM175 pathway. J Transl Med. (2024) 22:457. doi: 10.1186/s12967-024-05228-1, PMID: 38745204 PMC11095009

[166] Ge H Si L Li C Huang J Sun L Wu L. The antidepressant effect of resveratrol may correlate with the anti-inflammatory pathways mediated by Tas2r123 in hippocampus. Int Immunopharmacol. (2025) 156:114670. doi: 10.1016/j.intimp.2025.114670, PMID: 40253765

[167] Wei RM Zhang YM Feng YZ Zhang KX Zhang JY Chen J. Resveratrol ameliorates maternal separation-induced anxiety- and depression-like behaviors and reduces Sirt1-NF-kB signaling-mediated neuroinflammation. Front Behav Neurosci. (2023) 17:1172091. doi: 10.3389/fnbeh.2023.1172091, PMID: 37273278 PMC10233157

[168] Shaito A Posadino AM Younes N Hasan H Halabi S Alhababi D. Potential adverse effects of resveratrol: A literature review. Int J Mol Sci. (2020) 21:2084. doi: 10.3390/ijms21062084, PMID: 32197410 PMC7139620

[169] Wang J Men Y Wang Z. Polydatin alleviates chronic stress-induced depressive and anxiety-like behaviors in a mouse model. ACS Chem Neurosci. (2023) 14:977–87. doi: 10.1021/acschemneuro.2c00758, PMID: 36802487

[170] Zhang N Ma Y Li Y Wang Y Zhang L Zheng M. Paeonol prevents sepsis-associated encephalopathy via regulating the HIF1A pathway in microglia. Int Immunopharmacol. (2024) 143:113287. doi: 10.1016/j.intimp.2024.113287, PMID: 39362015

[171] Chen P Guo Z Lei J Wang Y. Pomegranate polyphenol punicalin ameliorates lipopolysaccharide-induced memory impairment, behavioral disorders, oxidative stress, and neuroinflammation via inhibition of TLR4-NF-кB pathway. Phytother Res. (2024) 38:3489–508. doi: 10.1002/ptr.8219, PMID: 38695373

[172] Liu Y Dai C Wang C Wang J Yan W Luo M. Raspberry ketone prevents LPS-induced depression-like behaviors in mice by inhibiting TLR-4/NF-κB signaling pathway via the gut-brain axis. Mol Nutr Food Res. (2024) 68:e2400090. doi: 10.1002/mnfr.202400090, PMID: 38757671

[173] Tao W Hu Y Chen Z Dai Y Hu Y Qi M. Magnolol attenuates depressive-like behaviors by polarizing microglia towards the M2 phenotype through the regulation of Nrf2/HO-1/NLRP3 signaling pathway. Phytomedicine. (2021) 91:153692. doi: 10.1016/j.phymed.2021.153692, PMID: 34411834

[174] Zhang J Li L Liu Q Zhao Z Su D Xiao C. Gastrodin programs an Arg-1+ microglial phenotype in hippocampus to ameliorate depression- and anxiety-like behaviors via the Nrf2 pathway in mice. Phytomedicine. (2023) 113:154725. doi: 10.1016/j.phymed.2023.154725, PMID: 36867963

[175] Carrasco M Guzman L Olloquequi J Cano A Fortuna A Vazquez-Carrera M. Licochalcone A prevents cognitive decline in a lipopolysaccharide-induced neuroinflammation mice model. Mol Med. (2025) 31:54. doi: 10.1186/s10020-025-01106-8, PMID: 39930360 PMC11812219

[176] Zhang L He S Liu L Huang J. Saponin monomers: Potential candidates for the treatment of type 2 diabetes mellitus and its complications. Phytother Res. (2024) 38:3564–82. doi: 10.1002/ptr.8229, PMID: 38715375

[177] Liu Q Zhang J Xiao C Su D Li L Yang C. Akebia saponin D protects hippocampal neurogenesis from microglia-mediated inflammation and ameliorates depressive-like behaviors and cognitive impairment in mice through the PI3K-Akt pathway. Front Pharmacol. (2022) 13:927419. doi: 10.3389/fphar.2022.927419, PMID: 36110522 PMC9468712

[178] Lan Y Wang X Yan F Zhang W Zhao S Song Y. Quinoa saponin ameliorates lipopolysaccharide-induced behavioral disorders in mice by inhibiting neuroinflammation, modulating gut microbiota, and counterbalancing intestinal inflammation. J Agric Food Chem. (2025) 73:4700–15. doi: 10.1021/acs.jafc.5c00296, PMID: 39948027

[179] Chen S Wang K Wang H Gao Y Nie K Jiang X. The therapeutic effects of saikosaponins on depression through the modulation of neuroplasticity: From molecular mechanisms to potential clinical applications. Pharmacol Res. (2024) 201:107090. doi: 10.1016/j.phrs.2024.107090, PMID: 38309381

[180] Bi Y Li M Wang Y Yao J Wang Y Wang S. Saikosaponins from Bupleurum scorzonerifolium Willd. alleviates microglial pyroptosis in depression by binding and inhibiting P2X7 expression. Phytomedicine. (2025) 136:156240. doi: 10.1016/j.phymed.2024.156240, PMID: 39637473

[181] Wang M Li H Zhang W Zhang L Wang S Jia M. Saikosaponin A alleviates depressive-like behavior induced by reserpine in mice by regulating gut microflora and inflammatory responses. PloS One. (2025) 20:e0311207. doi: 10.1371/journal.pone.0311207, PMID: 39928658 PMC11809902

[182] Wang X Li S Yu J Wang W Du Z Gao S. Saikosaponin B2 ameliorates depression-induced microglia activation by inhibiting ferroptosis-mediated neuroinflammation and ER stress. J Ethnopharmacol. (2023) 316:116729. doi: 10.1016/j.jep.2023.116729, PMID: 37277081

[183] Bai Z Gao T Zhang R Lu Y Tian J Wang T. Inhibition of IL-6 methylation by Saikosaponin C regulates neuroinflammation to alleviate depression. Int Immunopharmacol. (2023) 118:110043. doi: 10.1016/j.intimp.2023.110043, PMID: 36965369

[184] Li X Li X Huang N Liu R Sun R. A comprehensive review and perspectives on pharmacology and toxicology of saikosaponins. Phytomedicine. (2018) 50:73–87. doi: 10.1016/j.phymed.2018.09.174, PMID: 30466994 PMC7126585

[185] Guo M Pei WJ Liu L Chen K Cheng Y Piao XL. Neuroprotective effects of gypenosides on LPS-induced anxiety and depression-like behaviors. Int Immunopharmacol. (2024) 143:113367. doi: 10.1016/j.intimp.2024.113367, PMID: 39413644

[186] Jiang Y Cheng X Zhao M Zhao T Zhang M Shi Z. Gypenoside-14 reduces depression via downregulation of the nuclear factor kappa B (NF-kB) signaling pathway on the lipopolysaccharide (LPS)-induced depression model. Pharm (Basel). (2023) 16:1152. doi: 10.3390/ph16081152, PMID: 37631068 PMC10459727

[187] Li J Zhao J Wang X Lin Z Lin H Lin Z. Ginsenoside - a promising natural active ingredient with steroidal hormone activity. Food Funct. (2024) 15:1825–39. doi: 10.1039/d3fo05484e, PMID: 38315542

[188] Li Y Li J Yang L Ren F Dong K Zhao Z. Ginsenoside Rb1 protects hippocampal neurons in depressed rats based on mitophagy-regulated astrocytic pyroptosis. Phytomedicine. (2023) 121:155083. doi: 10.1016/j.phymed.2023.155083, PMID: 37722244

[189] Kwon D Kim Y Cho SH. Antidepressant effects of ginsenoside rc on L-alpha-aminoadipic acid-induced astrocytic ablation and neuroinflammation in mice. Int J Mol Sci. (2024) 25:9673. doi: 10.3390/ijms25179673, PMID: 39273621 PMC11396248

[190] Chen H Dong M He H Piao X Han X Li R. Ginsenoside re prevents depression-like behaviors via inhibition of inflammation, oxidative stress, and activating BDNF/trkB/ERK/CREB signaling: an *in vivo* and *in vitro* study. J Agric Food Chem. (2024) 72:19838–51. doi: 10.1021/acs.jafc.4c04394, PMID: 39186472

[191] Li Z Li Y Liu C Gu Y Han G. Research progress of the mechanisms and applications of ginsenosides in promoting bone formation. Phytomedicine. (2024) 129:155604. doi: 10.1016/j.phymed.2024.155604, PMID: 38614042

[192] Zhang ZW Gao CS Zhang H Yang J Wang YP Pan LB. Morinda officinalis oligosaccharides increase serotonin in the brain and ameliorate depression via promoting 5-hydroxytryptophan production in the gut microbiota. Acta Pharm Sin B. (2022) 12:3298–312. doi: 10.1016/j.apsb.2022.02.032, PMID: 35967282 PMC9366226

[193] Li Z Xu H Xu Y Lu G Peng Q Chen J. Morinda officinalis oligosaccharides alleviate depressive-like behaviors in post-stroke rats via suppressing NLRP3 inflammasome to inhibit hippocampal inflammation. CNS Neurosci Ther. (2021) 27:1570–86. doi: 10.1111/cns.13732, PMID: 34559953 PMC8611777

[194] Zhu ZH Yin XY Xu TS Tao WW Yao GD Wang PJ. Morinda officinalis oligosaccharides mitigate chronic mild stress-induced inflammation and depression-like behavior by deactivating the MyD88/PI3K pathway via E2F2. Front Pharmacol. (2022) 13:855964. doi: 10.3389/fphar.2022.855964, PMID: 36052143 PMC9426723

[195] Yang L Ao Y Li Y Dai B Li J Duan W. Morinda officinalis oligosaccharides mitigate depression-like behaviors in hypertension rats by regulating Mfn2-mediated mitophagy. J Neuroinflammation. (2023) 20:31. doi: 10.1186/s12974-023-02715-y, PMID: 36765376 PMC9912533

[196] Wang M Sun P Li Z Li J Lv X Chen S. Eucommiae cortex polysaccharides attenuate gut microbiota dysbiosis and neuroinflammation in mice exposed to chronic unpredictable mild stress: Beneficial in ameliorating depressive-like behaviors. J Affect Disord. (2023) 334:278–92. doi: 10.1016/j.jad.2023.04.117, PMID: 37156274

[197] Qin YQ Wang LY Yang XY Xu YJ Fan G Fan YG. Inulin: properties and health benefits. Food Funct. (2023) 14:2948–68. doi: 10.1039/d2fo01096h, PMID: 36876591

[198] Wang L Wang Z Lan Y Tuo Y Ma S Liu X. Inulin attenuates blood-brain barrier permeability and alleviates behavioral disorders by modulating the TLR4/myD88/NF-κB pathway in mice with chronic stress. J Agric Food Chem. (2023) 71:13325–37. doi: 10.1021/acs.jafc.3c03568, PMID: 37642581

[199] Chang L Wang C Peng J Song Y Zhang W Chen Y. Rattan pepper polysaccharide regulates DSS-induced intestinal inflammation and depressive behavior through microbiota-gut-brain axis. J Agric Food Chem. (2024) 72:437–48. doi: 10.1021/acs.jafc.3c08462, PMID: 38164789

[200] Olofinsan K Abrahamse H George BP. Therapeutic role of alkaloids and alkaloid derivatives in cancer management. Molecules. (2023) 28:5578. doi: 10.3390/molecules28145578, PMID: 37513450 PMC10386240

[201] Wen J Li L Ou D Li J Yang Y Duan L. Higenamine protects against doxorubicin-induced heart failure by attenuating ferroptosis via modulating the Nrf2/GPX4 signaling pathway. Phytomedicine. (2025) 141:156670. doi: 10.1016/j.phymed.2025.156670, PMID: 40220414

[202] Yao J Chen C Sun Y Lin Y Tian Z Liu X. Higenamine exerts antidepressant effect by improving the astrocytic gap junctions and inflammatory response. J Affect Disord. (2024) 348:107–15. doi: 10.1016/j.jad.2023.12.020, PMID: 38101523

[203] Xu M Li W Hu X Zhang J. Arecoline alleviates depression via gut-brain axis modulation, neurotransmitter balance, neuroplasticity enhancement, and inflammation reduction in CUMS mice. J Agric Food Chem. (2025) 73:10201–13. doi: 10.1021/acs.jafc.4c11643, PMID: 40257350

[204] Zhang M Li A Yang Q Li J Zheng L Wang G. Matrine alleviates depressive-like behaviors via modulating microbiota-gut-brain axis in CUMS-induced mice. J Transl Med. (2023) 21:145. doi: 10.1186/s12967-023-03993-z, PMID: 36829227 PMC9951532

[205] Xu HW Li WF Hong SS Shao JJ Chen JH Chattipakorn N. Tabersonine, a natural NLRP3 inhibitor, suppresses inflammasome activation in macrophages and attenuate NLRP3-driven diseases in mice. Acta Pharmacol Sin. (2023) 44:1252–61. doi: 10.1038/s41401-022-01040-z, PMID: 36627344 PMC10203108

[206] Shi Y Hu Y Gan Y Mi Z Luo S Lei J. Tabersonine ameliorates depressive-like behavior by inhibiting NLRP3 inflammasome activation in a mouse model. Neuropharmacology. (2025) 273:110432. doi: 10.1016/j.neuropharm.2025.110432, PMID: 40147640

[207] Feng X Sureda A Jafari S Memariani Z Tewari D Annunziata G. Berberine in cardiovascular and metabolic diseases: from mechanisms to therapeutics. Theranostics. (2019) 9:1923–51. doi: 10.7150/thno.30787, PMID: 31037148 PMC6485276

[208] Yang L Huang Y Chen F Wang Y Su K Zhao M. Berberine attenuates depression-like behavior by modulating the hippocampal NLRP3 ubiquitination signaling pathway through Trim65. Int Immunopharmacol. (2023) 123:110808. doi: 10.1016/j.intimp.2023.110808, PMID: 37595491

[209] Qin Z Shi DD Li W Cheng D Zhang YD Zhang S. Berberine ameliorates depression-like behaviors in mice via inhibiting NLRP3 inflammasome-mediated neuroinflammation and preventing neuroplasticity disruption. J Neuroinflammation. (2023) 20:54. doi: 10.1186/s12974-023-02744-7, PMID: 36859349 PMC9976521

[210] Imenshahidi M Hosseinzadeh H. Berberine and barberry (Berberis vulgaris): A clinical review. Phytother Res. (2019) 33:504–23. doi: 10.1002/ptr.6252, PMID: 30637820

[211] Yang ZJ Huang SY Zhong KY Huang WG Huang ZH He TT. Betaine alleviates cognitive impairment induced by homocysteine through attenuating NLRP3-mediated microglial pyroptosis in an m6A-YTHDF2-dependent manner. Redox Biol. (2024) 69:103026. doi: 10.1016/j.redox.2024.103026, PMID: 38184996 PMC10808937

[212] Hui R Xu J Zhou M Xie B Zhou M Zhang L. Betaine improves METH-induced depressive-like behavior and cognitive impairment by alleviating neuroinflammation via NLRP3 inflammasome inhibition. Prog Neuropsychopharmacol Biol Psychiatry. (2024) 135:111093. doi: 10.1016/j.pnpbp.2024.111093, PMID: 39029648

[213] Liang Y Chen L Huang Y Xie L Liu X Zhou W. Betaine eliminates CFA-induced depressive-like behavior in mice may be through inhibition of microglia and astrocyte activation and polarization. Brain Res Bull. (2024) 206:110863. doi: 10.1016/j.brainresbull.2023.110863, PMID: 38145759

[214] Li Z He X Liu F Wang J Feng J. A review of polysaccharides from Schisandra chinensis and Schisandra sphenanthera: Properties, functions and applications. Carbohydr Polym. (2018) 184:178–90. doi: 10.1016/j.carbpol.2017.12.058, PMID: 29352909

[215] Yang Y Liu R Sun Y Wu B He B Jia Y. Schisandrin B restores M1/M2 balance through miR-124 in lipopolysaccharide-induced BV2 cells. J Pharm Pharmacol. (2024) 76:1352–61. doi: 10.1093/jpp/rgae079, PMID: 39024474

[216] Wang J Zhang G Yang Y Zhang X Shi K Zhang X. Schisandra chinensis Lignans Exert Antidepressant Effects by Promoting BV2 Microglia Polarization toward the M2 Phenotype through the Activation of the Cannabinoid Receptor Type-2-Signal Transducer and Activator of Transcription 6 Pathway. J Agric Food Chem. (2022) 70:14157–69. doi: 10.1021/acs.jafc.2c04565, PMID: 36349542

[217] Jin GN Wang Y Liu YM Lu YN Lu JM Wang JH. Arctiin mitigates neuronal injury by modulating the P2X7R/NLPR3 inflammasome signaling pathway. Inflammation. (2025) 48(3):987–1002. doi: 10.1007/s10753-024-02117-z, PMID: 39154088

[218] Chen PL Xu GH Li M Zhang JY Cheng J Li CF. Yamogenin exhibits antidepressant-like effects via inhibition of ER stress and microglial activation in LPS-induced mice. ACS Chem Neurosci. (2023) 14:3173–82. doi: 10.1021/acschemneuro.3c00306, PMID: 37579249

[219] Tao X Zhou Y Wang Z Wang L Xia T Yan M. Cajaninstilbene acid ameliorates depression-like behaviors in mice by suppressing TLR4/NF-κB mediated neuroinflammation and promoting autophagy. Behav Brain Res. (2024) 471:115142. doi: 10.1016/j.bbr.2024.115142, PMID: 38972486

[220] Huang L Wang S Zhang Q Zhang J Sun J. Fucosterol ameliorates depressive-like behaviors by suppressing microglial activation and neuroinflammation via inhibition the MAPK/ERK1/2 signaling pathway. Int Immunopharmacol. (2025) 158:114821. doi: 10.1016/j.intimp.2025.114821, PMID: 40349406

[221] Li C Cui Z Deng S Chen P Li X Yang H. The potential of plant extracts in cell therapy. Stem Cell Res Ther. (2022) 13:472. doi: 10.1186/s13287-022-03152-z, PMID: 36104798 PMC9476258

[222] Kim H Choi HS Han K Sim W Suh HJ Ahn Y. Ashwagandha (Withania somnifera (L.) dunal) root extract containing withanolide a alleviates depression-like behavior in mice by enhancing the brain-derived neurotrophic factor pathway under unexpected chronic mild stress. J Ethnopharmacol. (2025) 340:119224. doi: 10.1016/j.jep.2024.119224, PMID: 39674356

[223] Shin J Kim DU Bae GS Han JY Lim DW Lee YM. Antidepressant-like effects of cannabis sativa L. Extract in an lipopolysaccharide model: modulation of mast cell activation in deep cervical lymph nodes and dura mater. Pharm (Basel). (2024) 17:1409. doi: 10.3390/ph17101409, PMID: 39459047 PMC11510560

[224] Xie Y Wu Z Qian Q Yang H Ma J Luan W. Apple polyphenol extract ameliorates sugary-diet-induced depression-like behaviors in male C57BL/6 mice by inhibiting the inflammation of the gut-brain axis. Food Funct. (2024) 15:2939–59. doi: 10.1039/d3fo04606k, PMID: 38406886

[225] Arshad HM Ahmad FU Lodhi AH. Methanolic extract of aerva javanica leaves prevents LPS-induced depressive like behavior in experimental mice. Drug Des Devel Ther. (2022) 16:4179–204. doi: 10.2147/DDDT.S383054, PMID: 36514526 PMC9741839

[226] Zhang X Li Q Ye X Chen Q Chen C Hu G. The impacts of natural product miltirone and the CYP2D6 pharmacogenetic phenotype on fluoxetine metabolism. Front Pharmacol. (2024) 15:1373048. doi: 10.3389/fphar.2024.1373048, PMID: 38741591 PMC11089247

[227] Guo C Huang Q Wang Y Yao Y Li J Chen J. Therapeutic application of natural products: NAD+ metabolism as potential target. Phytomedicine. (2023) 114:154768. doi: 10.1016/j.phymed.2023.154768, PMID: 36948143

